# Comparison of cytokine responses to group B *Streptococcus* infection in a human maternal-fetal interface organ-on-a-chip system and *ex vivo* culture model of human gestational membranes

**DOI:** 10.1128/iai.00346-25

**Published:** 2025-11-24

**Authors:** Leslie A. Kirk, Hannah A. Richards, Danyvid Olivares-Villagómez, Andrea Locke, Anthony R. Flores, Shannon D. Manning, David M. Aronoff, Kevin G. Osteen, David E. Cliffel, Alison J. Eastman, Jennifer A. Gaddy

**Affiliations:** 1 Department of Medicine, Division of Infectious Diseases, Vanderbilt University Medical Center 12328, Nashville, Tennessee, USA; 2 Department of Chemistry, Vanderbilt University 189509, Nashville, Tennessee, USA; 3 Department of Biomedical Engineering, Vanderbilt University 575178, Nashville, Tennessee, USA; 4 Department of Pediatrics, Division of Pediatric Infectious Diseases, Vanderbilt University Medical Center 12328, Nashville, Tennessee, USA; 5 Department of Microbiology, Genetics, and Immunology, Michigan State University 3078, East Lansing, Michigan, USA; 6 Department of Medicine, Indiana University School of Medicine 12250, Indianapolis, Indiana, USA; 7 Department of Obstetrics and Gynecology, Vanderbilt University Medical Center 12328, Nashville, Tennessee, USA; 8 Department of Pathology, Microbiology, and Immunology, Vanderbilt University Medical Center 204907, Nashville, Tennessee, USA; 9 Department of Veterans Affairs, Tennessee Valley Health Systems, Nashville, Nashville, USA; 10 Medicine Health and Society, Vanderbilt University 5718, Nashville, Tennessee, USA; University of California San Diego School of Medicine, La Jolla, California, USA

**Keywords:** gestational membranes, reproductive immunology, pregnancy, *Streptococcus*, bacteria

## Abstract

Adverse pregnancy outcomes represent a global health burden. Bacterial infection and subsequent inflammation in gestational membranes lead to immunological and physiological changes that contribute to adverse pregnancy outcomes. Although animal models of infection during pregnancy are useful to interrogate tissue and cellular level changes in host responses, these models also have numerous drawbacks, including cost, complexity, and ethical considerations. The advent of organ-on-a-chip models provides cutting-edge new approaches to model host-pathogen interactions in multicellular organ and tissue environments. In this work, we employ an organ-on-a-chip model of the maternal-fetal interface as a tool to study immunological responses to infection with the perinatal pathogen, Group B *Streptococcus* (GBS). Furthermore, we validate the organ-on-a-chip assays using an *ex vivo* culture model of primary human gestational membranes. GBS infection leads to enhanced production of EGF, FGF-2, G-CSF, GRO-α, IL-6, IL-8, MCP-1, MIP-1α, TNF-β, IL-10, IL-17F in gestational membranes and both the maternal and fetal chambers of the organ-on-a-chip model. Additionally, GBS infection is associated with enhanced TNF-α, RANTES, IL-12p70, IP-10, MIG, FLT3L, GM-CSF, IL-1β, IL-2, PDGF-AB/BB, and IL-17E/IL-25 cytokine production in gestational membranes and the maternal compartment of the organ-on-a-chip model. Gestational membranes challenged with GBS produce IL-15, IL-27, M-CSF, MCP-3, MDC, and MIP-1β, a result that was not seen in the organ-on-a-chip model. GBS infection leads to enhanced production of eotaxin, IFN-γ, IL-1α, IL-4, IL-12p40, IL-13, and SCD40L in the maternal and fetal chambers of the organ-on-a-chip model, but not the gestational membranes *ex vivo*. Together, these results indicate that GBS infection induces comparable production of a repertoire of cytokines and chemokines in both models, with some salient differences, underscoring the utility of these complementary approaches to study immunological responses to infection at the maternal-fetal interface.

## INTRODUCTION

A common driver of adverse pregnancy outcomes is ascending vaginal colonization of the gravid host (1). Rectovaginal colonization is a risk factor for increased maternal and neonatal morbidity and mortality. For example, vaginal infections during pregnancy can lead to endometritis, chorioamnionitis, intrauterine growth restriction, preterm prelabor rupture of membranes (PPROM), preterm birth, low birth weight, maternal and neonatal sepsis, neonatal meningitis, and, in severe cases, maternal demise, miscarriage, or stillbirth (2, 3).

Preterm birth, defined as parturition prior to 37 weeks gestation, is the leading contributor to neonatal demise in the world—accounting for 27% of all neonatal deaths (4). The World Health Organization estimates the global burden of preterm birth to be 15 million cases each year (5). Geographic distribution of preterm birth incidence is heterogeneous, with incidences of 5–10% in the European Union (6). The incidence of preterm birth is 12.7% in the United States, whereas other developed nations, including Japan, Sweden, New Zealand, and Australia, have rates between 4% and 8% (2, 7–9). This underscores the unique factors that likely impact rates of adverse pregnancy outcomes, such as preterm birth, across the globe.

One risk factor for adverse pregnancy outcomes, such as preterm birth, is rectovaginal colonization with *Streptococcus agalactiae* (also known as Group B *Streptococcus*, GBS) (3). GBS is an encapsulated gram-positive bacterium that can colonize the gastrointestinal or urogenital tract of a healthy human host (10). However, pregnancy results in anatomical and immunological changes that alter host susceptibility to invasive infection with this pathobiont (11). To combat this, a limited number of countries have adopted screening for rectovaginal colonization of GBS during pregnancy, and patients positive for GBS colonization are provided intrapartum antibiotics. However, the combination of emerging antibiotic resistance in clinical strains of GBS (12) and patient hypersensitivity to certain classes of antibiotics, such as first-line beta-lactam treatments (13), has challenged clinical management of these patients. Additionally, antibiotics disrupt the establishment of a healthy microbiome, which is increasingly appreciated as a crucial driver of health and development (14–17), highlighting the need for precision therapies to combat GBS-associated diseases, especially during pregnancy. Immunotherapeutics represent a new and expanding field of interest that could potentially be deployed at the maternal-fetal interface. However, there is a paucity of knowledge about the immunological responses to bacterial infection at the maternal-fetal interface.

Recent work indicates that infection during pregnancy induces proinflammatory signaling cascades that likely perturb the fragile tolerance between the maternal host and the semi-allogenic fetus (18, 19). In the case of GBS infection, our group as well as others has utilized a variety of cell and tissue models in tandem with animal models of infection to elucidate the immunological and physiological changes that occur in response to GBS infection at the maternal-fetal interface (20–23). However, these approaches have several caveats. For example, animal models of infection during pregnancy are time-consuming and expensive. Traditional cell culture models are limited to responses in single cell lines without the important context of interactions with other cells in a tissue or organ model. Human *ex vivo* tissue models have significant heterogeneity due to genetic and environmental differences from patient to patient, which introduce variability. Additionally, these tissues rely on human donors, which present cumbersome logistic and administrative challenges. Thus, there is a pressing need for scalable, reproducible, translational models to study immune responses to infection at the maternal-fetal interface.

 In this work, we present data demonstrating a maternal-fetal interface organ-on-a-chip system with a dual-chamber design that provides a strong platform to study maternal and fetal responses to infection in a single device with strong reproducible signals from several proinflammatory cytokines as determined by multiplex analysis. The maternal-fetal interface in humans is comprised of maternally derived decidual stromal cells, fetally derived cytotrophoblasts, and placental macrophages that patrol the tissue interface. In our novel maternal-fetal interface organ-on-a-chip system, we utilize one chamber for decidual stromal cells co-cultured with macrophages and the second chamber for cytotrophoblasts co-cultured with macrophages. The two chambers are separated by a gelatin matrix to facilitate paracrine signaling between the two chambers, and microfluidic ports are used to introduce GBS bacterial cells into the maternal and fetal compartments and to draw out supernatant fractions for immunophenotyping. Importantly, we validate this organ-on-a-chip platform using *ex vivo* gestational membranes as a model to study infection and find significant overlap in the responses between the organ-on-a-chip model and the *ex vivo* primary human tissue model, underscoring the utility of these complementary techniques to study infection of the maternal-fetal interface.

## MATERIALS AND METHODS

### Bacterial strains and culture conditions

These experiments utilized *Streptococcus agalactiae* strain GB00112 (GB112), a capsular type III (cpsIII) strain isolated from the recto-vaginal swab of a patient who had recently given birth and characterized by multilocus sequencing typing in 2008 (24), and strain GB00037 (GB37), an invasive capsular type V strain isolated from a case of neonatal sepsis (25). The aforementioned strains were selected because cpsIII strains are one of the leading causes of invasive neonatal GBS infections, but cpsV is an emerging strain type of concern (26). GBS strains were streaked onto tryptic soy agar plates with 5% sheep blood (blood agar) and incubated at 37°C overnight. Isolated colonies were picked and inoculated into Todd Hewitt Broth (THB, Thermo Fisher) and incubated in a shaking incubator at 200 RPM and at 37°C overnight.

### Human gestational membrane co-culture assays

Placental and extraplacental tissues were obtained from patients scheduled for term (37 week or above), healthy, non-laboring c-section deliveries at Vanderbilt University Medical Center (approved by VUMC Institutional Review Board, #181998), and then de-identified. Gestational membranes were cut away from the placenta, leaving at least a 1-inch margin, rinsed in warm PBS to remove blood, and used to prepare full-thickness tissue samples with 12 mm biopsy punches. Punches were allowed to rest overnight in 37°C, 5% CO_2_ incubator, suspended in medium in 12-well dishes with 1 mL per well RPMI 1640 medium supplemented with 1% antibiotic/antimycotic solution (ThermoFisher) and 10% charcoal-stripped fetal bovine serum (FBS, Gibco), referred to as complete RPMI. Gestational membranes were rinsed the next day in PBS and then infected with 1 × 10^6^ colony-forming units (CFU)/mL of GB112 or GB37 in warm complete RPMI without antibiotics. Infected cultures were incubated for 24 h in a 37°C, 5% CO_2_ incubator in 12-well dishes. Full-thickness gestational membranes are one-quarter to one-half inch thick and require 24 h for GBS to penetrate and interact with the cell types therein. After 24 h of infection, supernatants were collected for cytokine analysis.

### Maternal-fetal interface organ-on-a-chip co-culture assays

These studies used the transformed human endometrial stromal cells (THESC) decidual stromal cell line (DSC, obtained from ATCC, Manassas, VA, USA), the HTR8 cytotrophoblast cell line (CTB, a kind gift from Dr. Vikki Abrahams, Yale University), and the THP-1 human monocytic leukemia-derived cell line (ATCC). These cell lines were chosen to model the maternal-fetal interface because the fetal membranes at this interface are composed of maternally derived DSCs, fetally derived CTB, and placental macrophage cells that patrol and surveil the tissue. DSCs were cultured in DMEM/F12 without phenol red (Invitrogen, Carlsbad, CA) supplemented with 10% charcoal-stripped fetal bovine serum (csFBS) and 1% antibiotic antimycotic solution (A/A) (Gibco, Waltham, Massachusetts, USA). DSCs were decidualized over a course of seven or more days treating every other day with 0.5 mM 8-Br-cAMP, 10 nM estradiol, and 1 µM medroxyprogesterone acetate (27, 28) (decidualization medium) (Sigma-Aldrich, St. Louis, MS, USA). Induction of prolactin and IGFBP1 secretion, indicative of decidualization, was confirmed by ELISA (Alpha Diagnostic International, San Antonio, TX, USA). CTBs were cultured in DMEM without phenol red (Invitrogen) supplemented with 10% csFBS and A/A. THP-1 cells were cultured in RPMI 1640 (Invitrogen) with 10% csFBS and A/A. THP-1 cells were differentiated into adherent macrophage-like cells by overnight treatment with phorbol myristate acetate (PMA, 5 ng/mL; Sigma, St. Louis, MO, USA). Nonadherent cells were washed off, and macrophages were removed by incubating adherent cells with Cell Dissociation Buffer Enzyme-Free PBS-based Gibco for 5 min at 37°C, followed by scraping with a cell scraper.

### Fabricating OoC

Molds were designed with Fusion 360 (Autodesk), 3D printed in resin using a Form 3 SLA 3D printer (FormaLabs), and then coated in the hydrophobic polymer parylene as described in O’Grady et al. (29). The mold is filled with Krayden Dow Sylgard 184 Silicone Elastomer Kit, clear (polydimethyl siloxane [PDMS], Fisher, Hampton, NH, USA), and gas in the mixed PDMS is removed in a vacuum chamber for 1 h at roughly −0.07 RoHS. The mold was placed in a heated chamber to polymerize at 80°C for 2 h or at 65°C overnight. The cast was removed from the mold, edges trimmed, and surface particulate removed using adhesive tape. It was placed active side up on a non-stick surface inside the plasma cleaner. A clean, large-format microscope slide coverslip (Ted Pella Inc, Cat# 260461-100, Redding, CA, USA) was also placed into the plasma cleaner. Plasma was initiated for 30 sec to 2 min. The chamber was opened, and the active side of the PDMS is pressed firmly onto the active side of the microscope coverslip. Finished devices were autoclaved on gravity cycle for 30 min.

### Preparing OoC

Ten percent gelatin was prepared by dissolving 1 g gelatin (Sigma) into 10 mL DMEM/F12 medium without antibiotics, heating at 65°C in a water bath until gelatin was dissolved, quickly sterilizing with a 0.2 µM syringe filter into a sterile conical, and keeping at 65°C until use. 100 mg/mL microbial transglutaminase (MTG, Moo Gloo, Modernist Pantry, Eliot, ME, USA) in PBS was sterilized with 0.2 µM syringe filter. 1:10 mixtures of MTG:gelatin were prepared immediately before use and pipetted into the central chamber using a P1000 pipette. Gelatin-loaded devices were put into a 37°C incubator overnight for gelatin to polymerize. Chambers were filled with poly-L-lysine (PLL, Sigma) and stored overnight at 37°C. Chambers were flushed and then refilled with collagen IV solution (Rockland Immunochemicals, Pottstown, PA, USA), diluted in 0.1N acetic acid, and stored overnight at 37°C. The chambers were flushed with PBS and then stored full of PBS at 37°C until use.

### Seeding, infecting, and harvesting OoC

We removed the PBS from one chamber and loaded it with THESC cells (0.5 × 10^6^ cells/mL). Cells were allowed to adhere overnight, and the medium was removed and replaced with decidualization medium on days 0, 2, 4, 6, and 8. On day 8, the second chamber was flushed and seeded with CTBs at 0.5 × 10^6^ cells/mL. Cells were allowed to adhere overnight. On day 9, macrophages were added to both chambers (0.5 × 10^5^ cells/mL) and allowed to settle for 1 h. The medium was removed, and GB112 or GB37 bacterial cells were diluted in DMEM/F12 with 10% csFBS, or uninfected control medium (both antibiotic-free), were added to both chambers. GBS was used at a multiplicity of infection (MOI) of 10 for DSC and CTB chambers, corresponding to an MOI of 100 relative to the THP-1 cells in the OoC, as THP-1 cells were used at a 1:10 ratio of THP-1 cells:DSC or CTBs. Infections were allowed to proceed for 1 h, after which medium was replaced with antibiotic-containing medium at 1× antibiotic concentration. The organ-on-a-chip device has a much smaller depth for cellular interactions, and long incubations result in cell death in these co-culture systems. To combat this, shorter time periods were utilized for the organ-on-a-chip devices and antibiotic treatment in order to limit bacteria-dependent cell death and prolong cell viability. The experiment was allowed to proceed for 24 h, and supernatants were collected for cytokine analysis.

### Multiplex cytokine analysis

Supernatants from punch biopsy cultures or OoC were frozen at −80°C until experiments concluded, then sent to Eve Technologies (Alberta, Canada) for multiplex cytokine array as previously described (30). Briefly, quantitative analysis of 48 cytokine targets was performed including soluble CD40 ligand (sCD40L), epidermal growth factor (EGF), eotaxin, fibroblast growth factor 2 (FGF-2), FMS-like tyrosine kinase 3 ligand (FLT-3), fractalkine, granulocyte colony-stimulating factor (G-CSF), granulocyte macrophage colony-stimulating factor (GM-CSF), growth-regulated oncogene α (GRO-α), interferon α 2 (IFNα2), interferon γ (IFNγ), interleukin (IL) 1α (IL-1α), IL-1β, IL-1RA, IL-2, IL-3, IL-4, IL-5, IL-6, IL-7, IL-8, IL-9, IL-10, IL-12p40, IL-12p70, IL-13, IL-15, IL-17A, IL-17E/IL-25, IL-17F, IL-18, IL-22, IL-27, interferon γ-induced protein 10 (IP-10), monocyte chemoattractant protein (MCP) 1 (MCP-1), MCP-3, macrophage colony-stimulating factor (M-CSF), macrophage-derived chemokine (MDC), monocyte-induced by interferon γ (MIG), macrophage inflammatory protein (MIP) 1α (MIP-1α), MIP-1β, platelet-derived growth factor (PDGF) AA (PDGF-AA), PDGF-AB/BB, regulated on activation, normal T-cell expressed and secreted (RANTES), transforming growth factor α (TGF-α), tumor necrosis factor (TNF) α (TNF-α), TNF-β, and vascular endothelial growth factor A (VEGF-A).

### Statistical analyses

Experiments were performed with three or more experimental replicates performed on different days with distinct batches of cells, tissues, and/or bacterial cultures. Statistical comparisons of parametric data between three or more experimental groups were performed by one-way ANOVA with Tukey’s post hoc multiple corrections test. *P* value < 0.05 was considered significant. Analysis was carried out using GraphPad Prism 10 (GraphPad Software Inc).

## RESULTS

### GBS infection leads to enhanced production of GRO-α, IL-6, and MIP-1α in gestational membranes and both the maternal and fetal chambers of the organ-on-a-chip model

**Fig 1:**
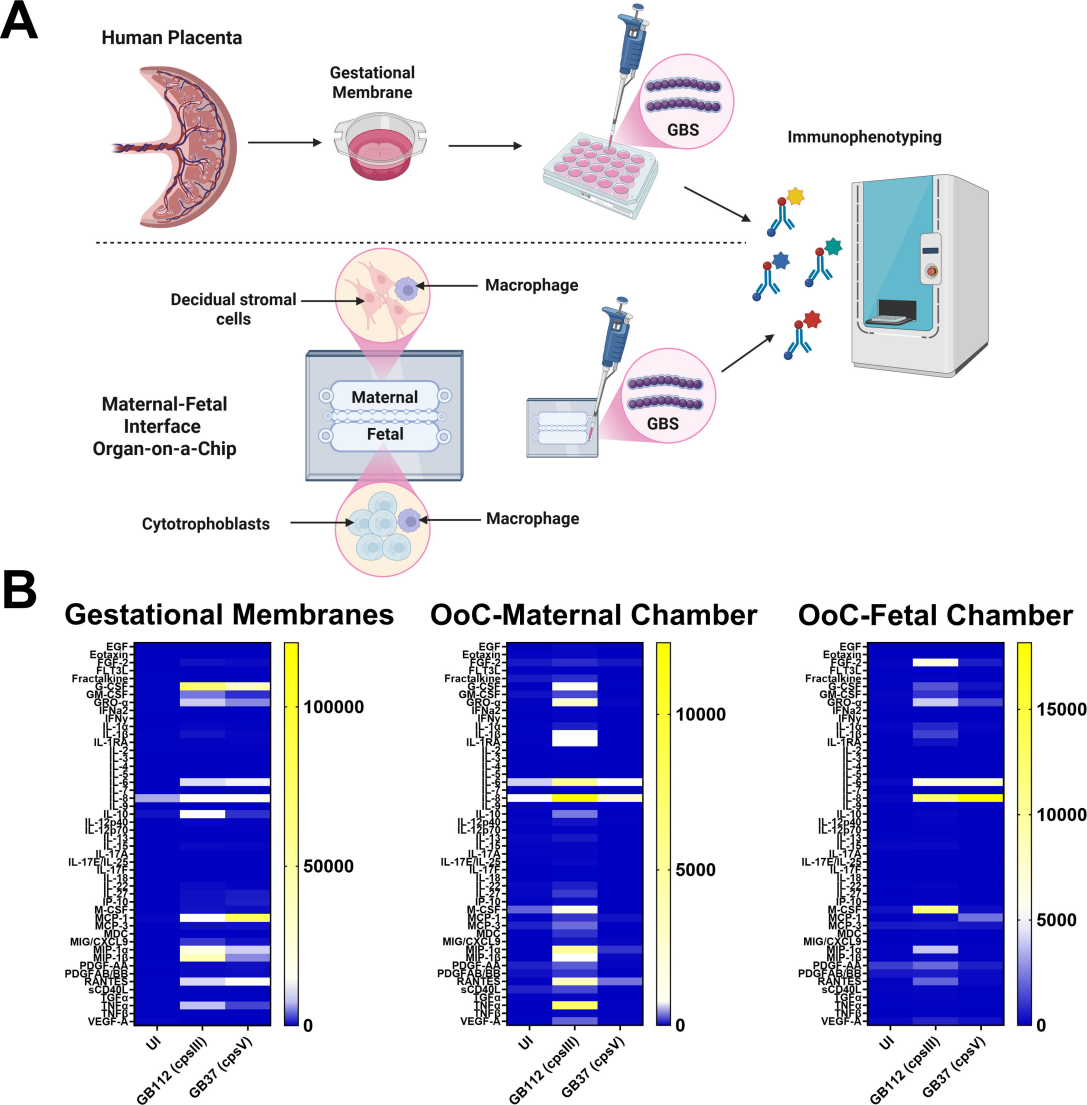
Human gestational membranes and maternal-fetal interface organ-on-a-chip (OoC) system responses to infection with the perinatal pathogen, Group B *Streptococcus* (GBS) strains GB112 (a capsular serotype III strain, cpsIII) or GB37 (a capsular serotype V strain, cpsV). (**A**) Conceptual diagram of the two models and approaches employed in this study. Human gestational membrane biopsies are taken from placenta derived from healthy, term, non-laboring C-section live births. The maternal-fetal interface organ-on-a-chip system consists of two compartments separated by a gelatin interface to facilitate paracrine signaling. The maternal compartment (OoC maternal chamber) contains decidual stromal cells and macrophages, and the fetal compartment (OoC fetal chamber) contains cytotrophoblasts and macrophages. Both models were infected with GBS, and uninfected (UI) controls were also maintained. Supernatants were collected and immunophenotyping was performed via multiplex cytokine analyses. (**B**) Heat map analysis of cytokine profiles (blue represents low cytokine concentrations, white represents moderate cytokine concentrations, and yellow represents high cytokine concentrations). Heat map intensity represents mean values calculated from *n* = 3 biological replicates.

**Fig 2:**
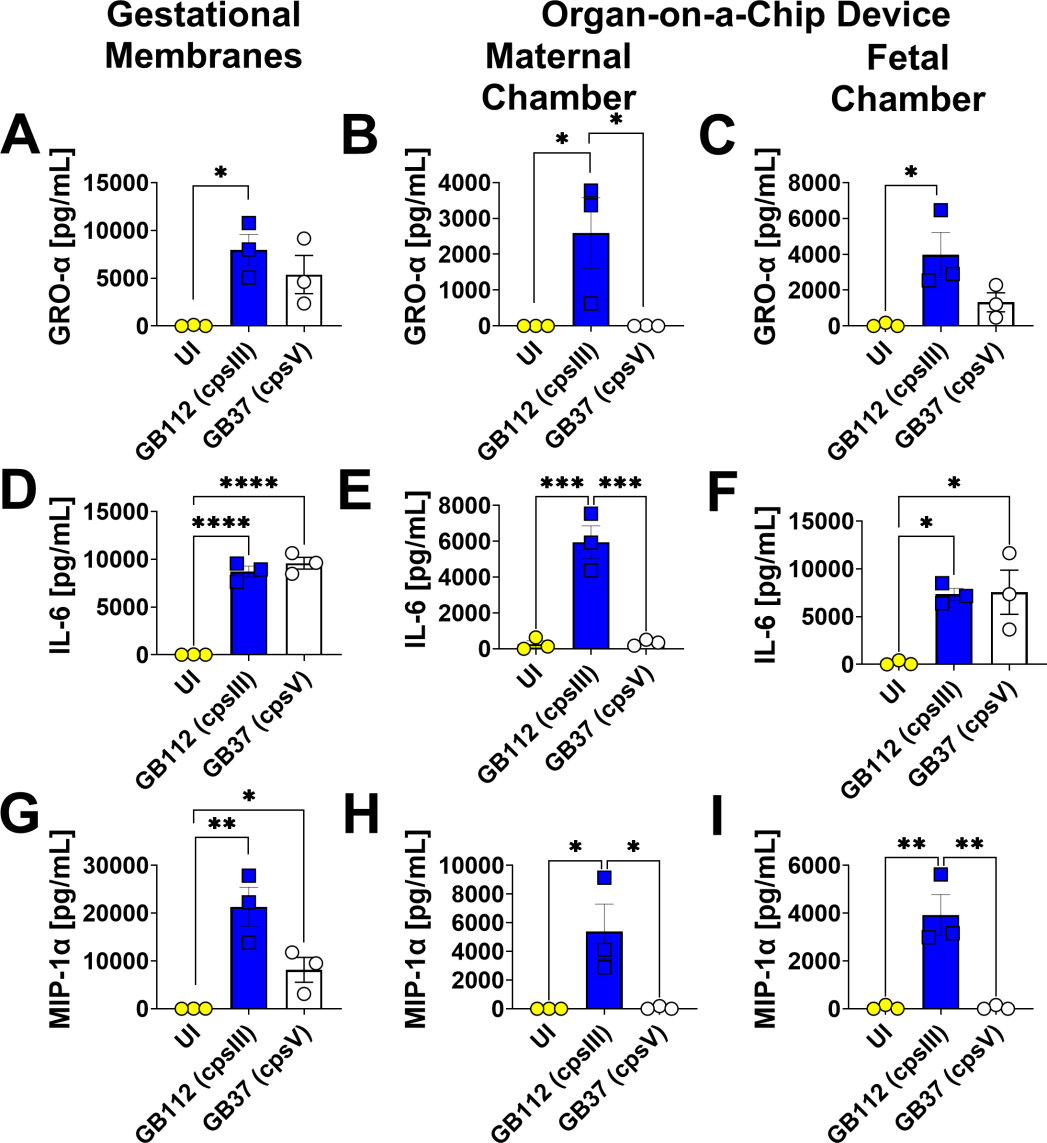
Analysis of GRO-α, IL-6, and MIP-1α production in human gestational membranes or maternal-fetal interface organ-on-a-chip (maternal or fetal chambers). Model systems were either maintained in uninfected conditions (UI, yellow circles and bars) or infected with the perinatal pathogen Group B *Streptococcus* (GB112, blue squares and bars; GB37, white circles and bars). Cytokines including GRO-α (**A–C**), IL-6 (**D–F**), and MIP-1α (**G–I**) were quantified by multiplex cytokine analyses. Gestational membranes (**A, D, and G**) are compared to the maternal chamber of (**B, E, and H**) or the fetal chamber (**C, F, and I**) the organ-on-a-chip device. Bars indicate mean values ± standard error mean error bars; individual points represent independent biological replicates. *P < 0.05, **P < 0.01, ***P < 0.001, ****P < 0.0001, one-way ANOVA with Tukey’s post hoc multiple corrections test. GRO-α, IL-6, and MIP-1α are significantly induced by GB112 infection in human gestational membranes and in both the maternal and fetal compartments of the organ-on-a-chip device.

In order to compare the utility of a maternal-fetal interface organ-on-a-chip system to an *ex vivo* human gestational membrane organ culture system, we employed multiplex cytokine analyses to compare these models in uninfected conditions or in the presence of infection with the perinatal pathogen GBS, including either a capsular serotype III strain (GB112, cpsIII) or a capsular serotype V strain (GB37, cpsV) (Fig. 1). Heat map analysis reveals differential cytokine production between uninfected and GBS-infected samples (Fig. 1). Specifically, infection with GBS resulted in a statistically significant increase in GRO-α, IL-6, and MIP-1α cytokine production in response to GB112 infection in gestational membranes, as well as in both the maternal and fetal chambers of the organ-on-a-chip model, compared to uninfected samples, as determined by one-way ANOVA with Tukey’s post hoc test (Fig. 2). GB37 infection significantly induced IL-6 and MIP-1α in gestational membranes and IL-6 in the fetal chamber of the organ-on-a-chip device compared to uninfected controls (Fig. 2); however, GB37 infection resulted in significantly less cytokine production compared to GB112 infection in several samples. Specifically, GB37 infection resulted in significantly less GRO-α in the maternal chamber, IL-6 in gestational membranes and the maternal chamber, and MIP-1α in the maternal and fetal chambers of the organ-on-a-chip device, compared to GB112 infected samples, as determined by one-way ANOVA with Tukey’s post hoc test (Fig. 2).

### GBS infection leads to enhanced production of G-CSF, GM-CSF, MCP-1, FLT3L, IL-1β, IL-8, TNF-α, TNF-β, 12p70, IL-17E, IL-17F, and PDGF-AB/BB in gestational membranes and the maternal chamber of the organ-on-a-chip model, but not the fetal chamber

**Fig 3:**
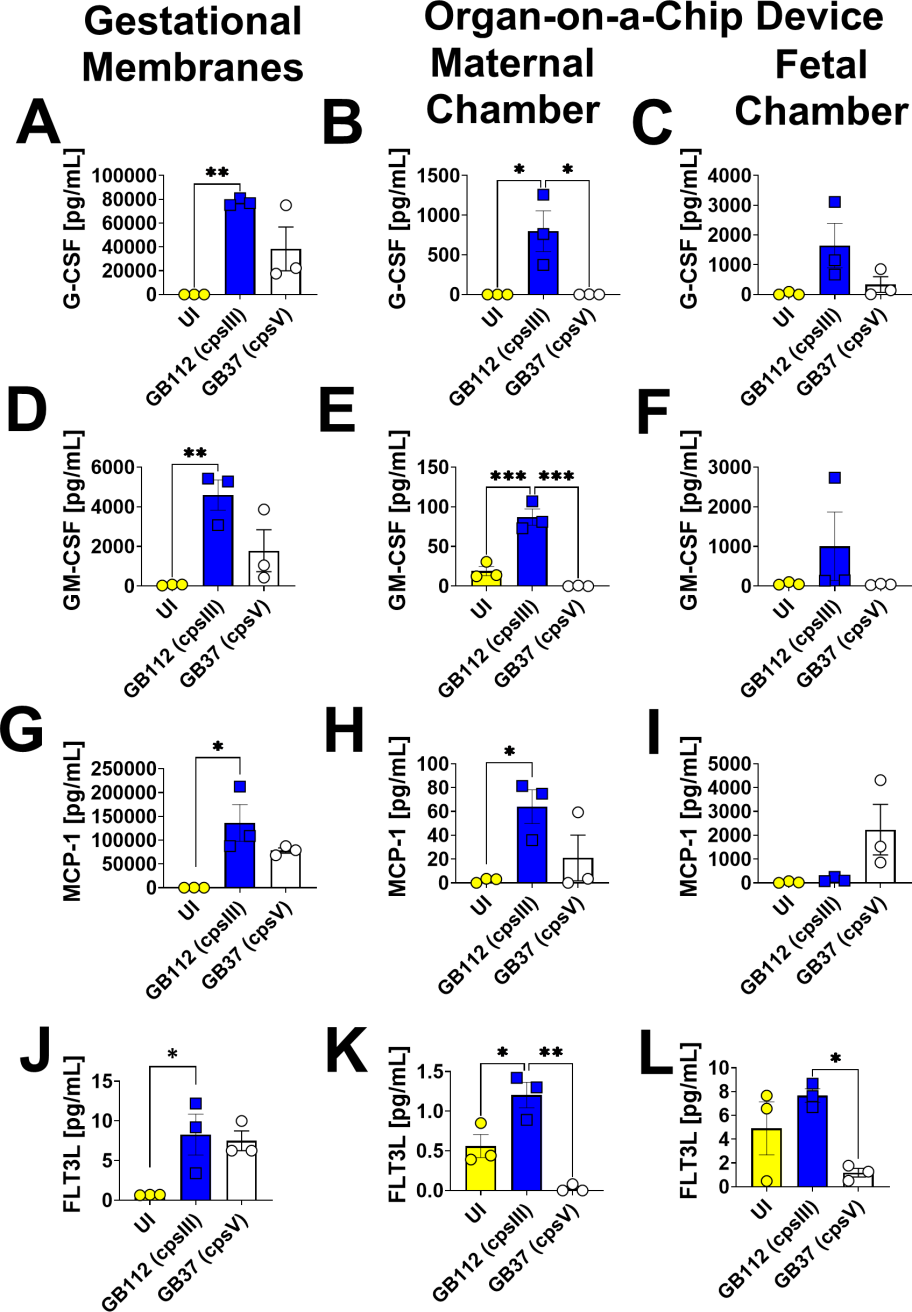
Analysis of G-CSF, GM-CSF, MCP-1, and FLT3L production in human gestational membranes or maternal-fetal interface organ-on-a-chip (maternal or fetal chambers). Model systems were either maintained in uninfected conditions (UI, yellow circles and bars) or infected with the perinatal pathogen Group B *Streptococcus* (GB112, blue squares and bars; GB37, white circles and bars). Cytokines including G-CSF (**A–C**), GM-CSF (**D–F**), MCP-1 (**G–I**), and FLT3L (**J–L**) were quantified by multiplex cytokine analyses. Gestational membranes (**A, D, G, and J**) are compared to the maternal chamber of (**B, E, H, and K**) or the fetal chamber (**C, F, I, and L**) the organ-on-a-chip device. Bars indicate mean values ± standard error mean error bars; individual points represent independent biological replicates. *P < 0.05, **P < 0.01, ***P < 0.001, ****P < 0.0001, one-way ANOVA with Tukey’s post hoc multiple corrections test. G-CSF, GM-CSF, MCP-1, and FLT3L are significantly induced by GB112 infection in human gestational membranes and in both the maternal and fetal compartments of the organ-on-a-chip device.

**Fig 4:**
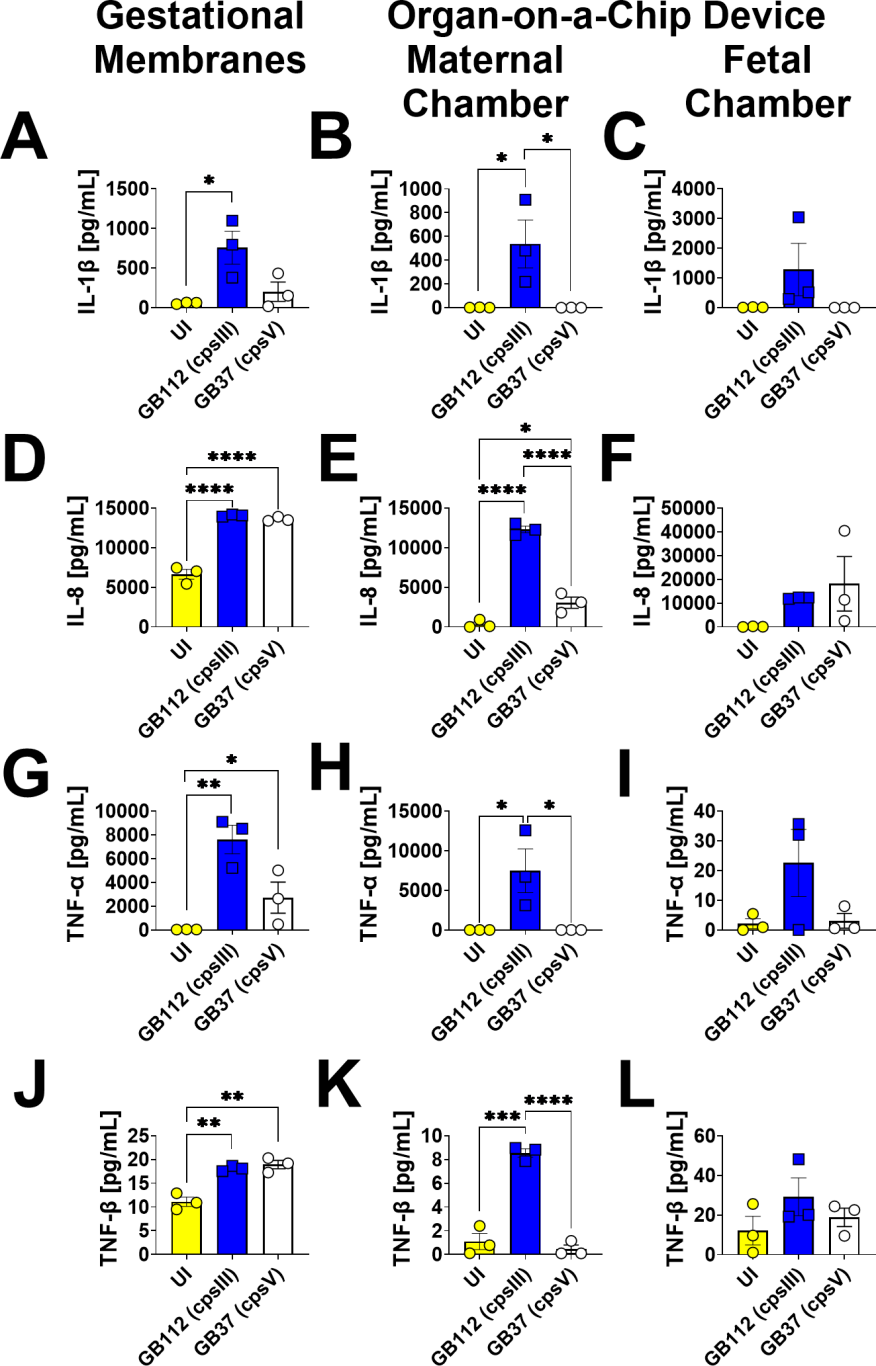
Analysis of IL-1β, IL-8, TNF-α, and TNF-β production in human gestational membranes or maternal-fetal interface organ-on-a-chip (maternal or fetal chambers). Model systems were either maintained in uninfected conditions (UI, yellow circles and bars) or infected with the perinatal pathogen Group B *Streptococcus* (GB112, blue squares and bars; GB37, white circles and bars). Cytokines including IL-1β (**A–C**), IL-8 (**D–F**), TNF-α (**G–I**), and TNF-β (**J–L**) were quantified by multiplex cytokine analyses. Gestational membranes (**A, D, and G**) are compared to the maternal chamber of (**B, E, and H**) or the fetal chamber (**C, F, and I**) the organ-on-a-chip device. Bars indicate mean values ± standard error mean error bars; individual points represent independent biological replicates. *P < 0.05, **P < 0.01, ***P < 0.001, ****P < 0.0001, one-way ANOVA with Tukey’s post hoc multiple corrections test IL-1β, IL-8, TNF-α, and TNF-β were significantly induced in gestational membranes and the maternal chamber, but not the fetal chamber of the organ-on-a-chip system in response to GB112 infection.

**Fig 5:**
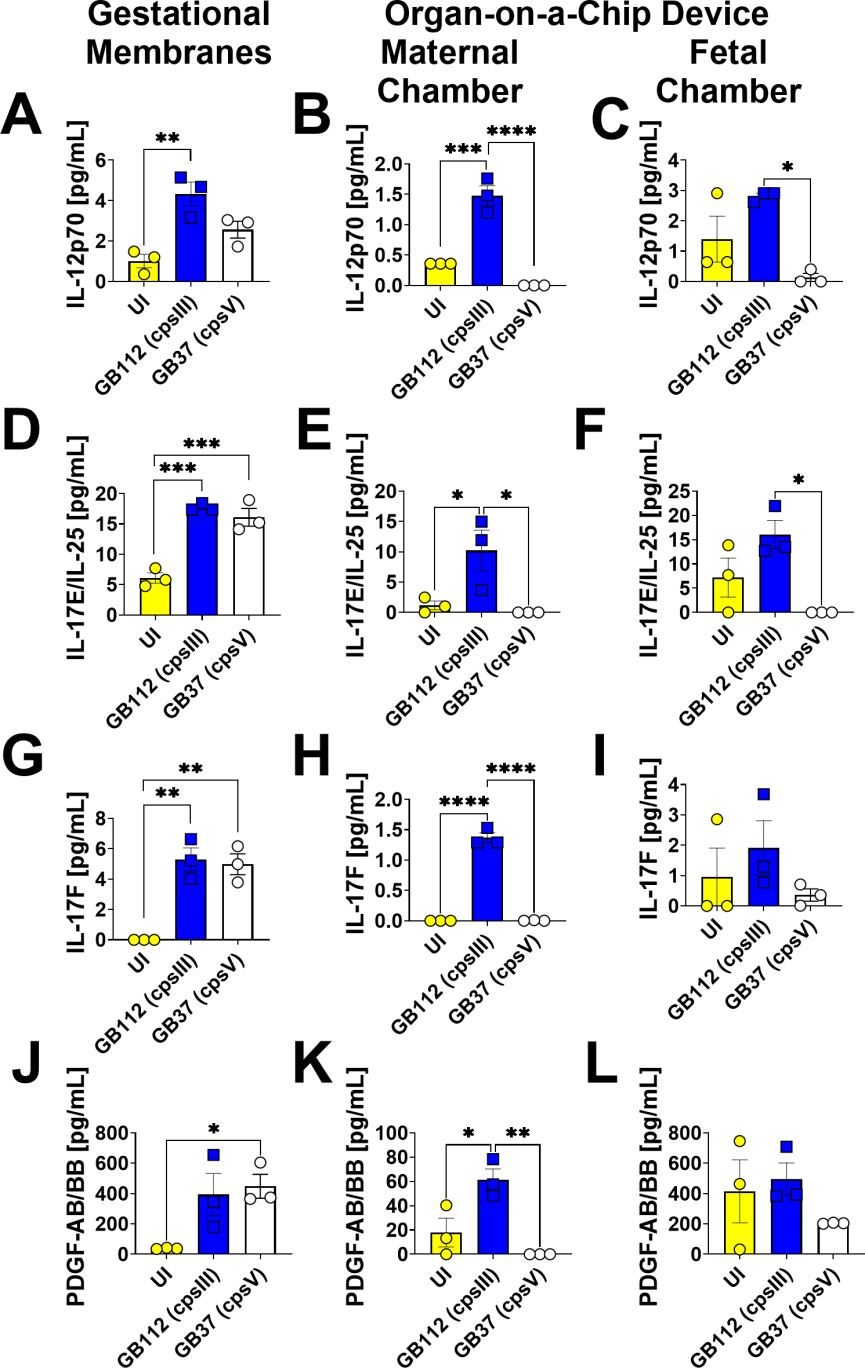
Analysis of IL-12p70, IL-17E/IL-25, IL-17F, and PDGF-AB/BB production in human gestational membranes or maternal-fetal interface organ-on-a-chip (maternal or fetal chambers). Model systems were either maintained in uninfected conditions (UI, yellow circles and bars) or infected with the perinatal pathogen Group B *Streptococcus* (GB112, blue squares and bars; GB37, white circles and bars). Cytokines including IL-12p70 (**A–C**), IL-17E/IL-25 (**D–F**), IL-17F (**G–I**), and PDGF-AB/BB (**J–L**) were quantified by multiplex cytokine analyses. Gestational membranes (**A, D, G, and J**) are compared to the maternal chamber of (**B, E, H, and K**) or the fetal chamber (**C, F, I, and L**) the organ-on-a-chip device. Bars indicate mean values ± standard error mean error bars; individual points represent independent biological replicates. *P < 0.05, **P < 0.01, ***P < 0.001, ****P < 0.0001, one-way ANOVA with Tukey’s post hoc multiple corrections test. IL-12p70, IL-17E/IL-25, IL-17F, and PDGF-AB/BB were significantly induced in gestational membranes and the maternal chamber, but not the fetal chamber of the organ-on-a-chip system in response to GB112 infection.

Infection with the capsular serotype III strain, GB112, resulted in the enhanced production of G-CSF, GM-CSF, MCP-1, FLT3, IL-1β, IL-8, TNF-α, TNF-β, 12p70, IL-17E, and IL-17F in primary human gestational membranes *ex vivo* as well as the maternal chamber of the organ-on-a-chip model (Fig. 3 to 5), as determined by one-way ANOVA with Tukey’s post hoc test. However, these cytokines were not significantly induced in the fetal chamber by GBS infection. Additionally, G-CSF, GM-CSF, MCP-1, FTL3L, IL-1β, and IL-12p70 cytokines were also not significantly induced by infection with the capsular serotype V strain, GB37, in gestational membranes or in the organ-on-a-chip models. Interestingly, PDGF-AB/BB was observed to be elevated in GB37-infected gestational membranes, but not in GB112-infected membranes. And conversely, PDGF-AB/BB was elevated in the maternal chamber of the organ-on-a-chip by GB112 infection, but not by GB37 infection (Fig. 5), underscoring the varying cell responses by different reproductive tract cell types to different strain types of GBS.

### GBS infection leads to enhanced production of EGF, IL-1α, IL-10, sCD40L, and fractalkine in the maternal and fetal chambers of the organ-on-a-chip model, but not human gestational membranes *ex vivo*

**Fig 6:**
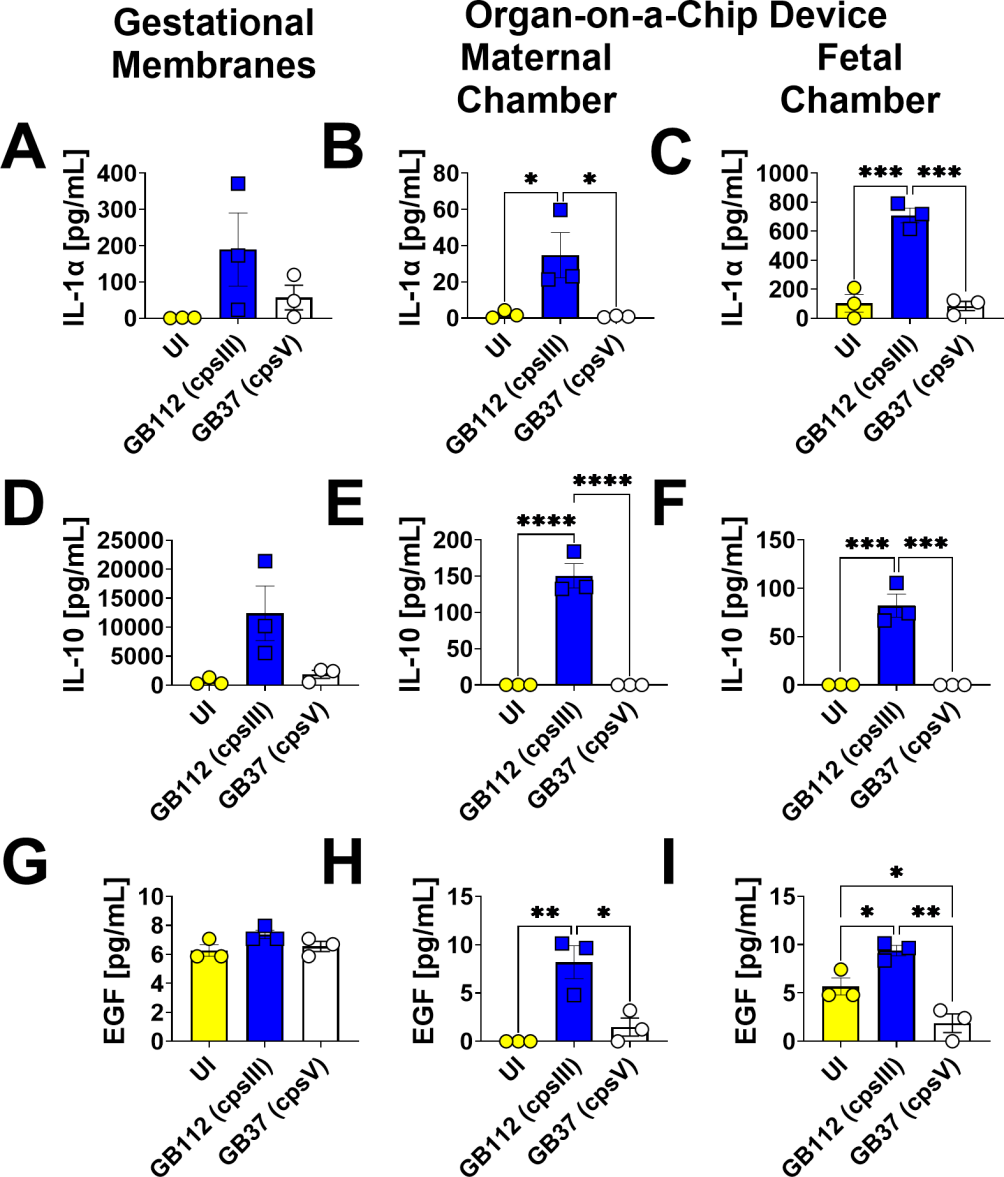
Analysis of IL-1α, IL-10, and EGF production in human gestational membranes or maternal-fetal interface organ-on-a-chip (maternal or fetal chambers). Model systems were either maintained in uninfected conditions (UI, yellow circles and bars) or infected with the perinatal pathogen Group B *Streptococcus* (GB112, blue squares and bars; GB37, white circles and bars). Cytokines including IL-1α (**A–C**), IL-10 (**D–F**), and EGF (**G–I**) were quantified by multiplex cytokine analyses. Gestational membranes (**A, D, and G**) are compared to the maternal chamber of (**B, E, and H**) or the fetal chamber (**C, F, and I**) the organ-on-a-chip device. Bars indicate mean values ± standard error mean error bars; individual points represent independent biological replicates. *P < 0.05, **P < 0.01, ***P < 0.001, ****P < 0.0001, one-way ANOVA with Tukey’s post hoc multiple corrections test. IL-1α, IL-10, and EGF were significantly induced in gestational membranes and the maternal chamber, but not the fetal chamber of the organ-on-a-chip system in response to GB112 infection.

**Fig 7:**
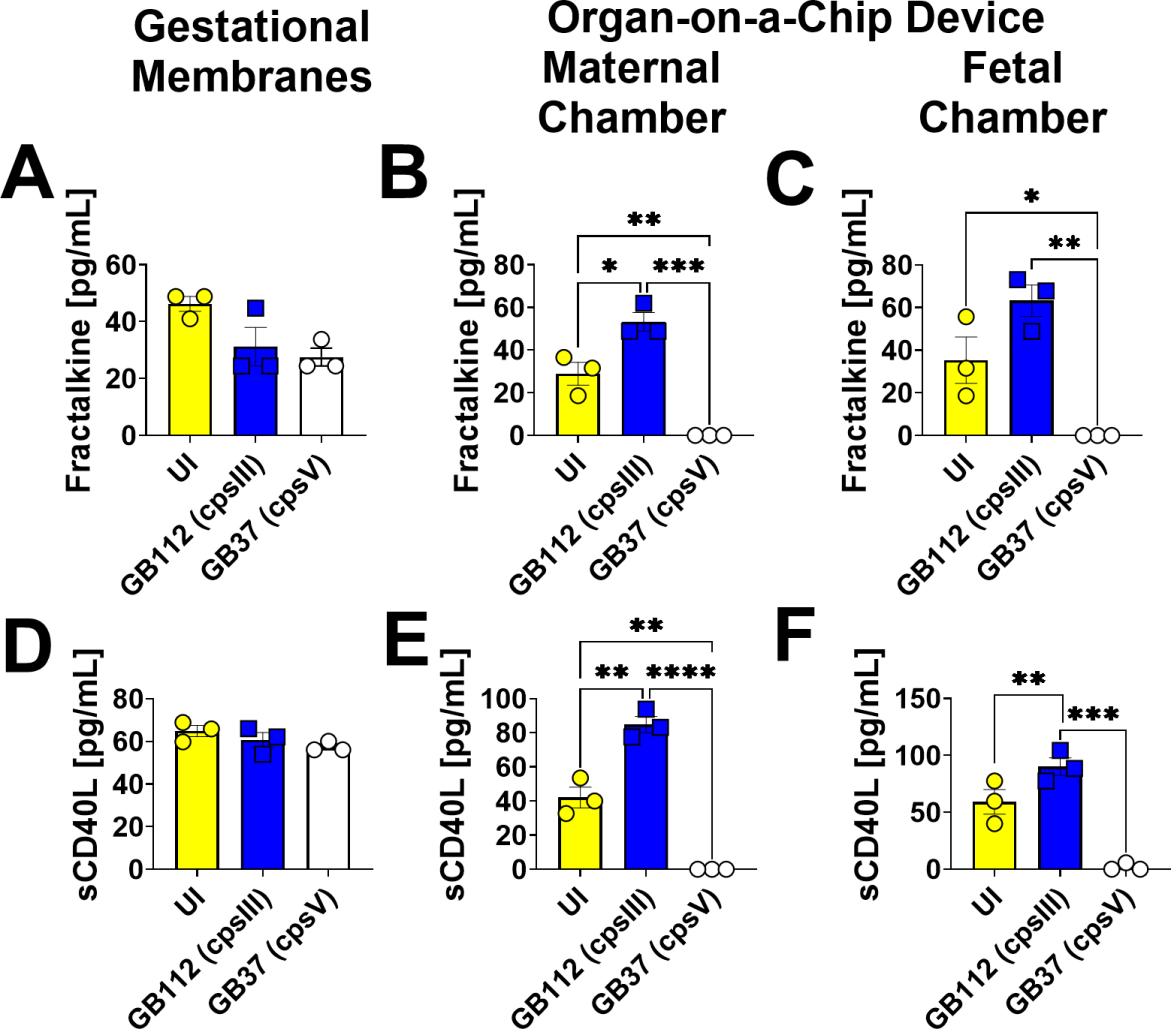
Analysis of fractalkine and sCD40L production in human gestational membranes or maternal-fetal interface organ-on-a-chip (maternal or fetal chambers). Model systems were either maintained in uninfected conditions (UI, yellow circles and bars) or infected with the perinatal pathogen Group B *Streptococcus* (GB112, blue squares and bars; GB37, white circles and bars). Cytokines including fractalkine (**A–C**) and sCD40L (**D–F**) were quantified by multiplex cytokine analyses. Gestational membranes (**A and D**) are compared to the maternal chamber of (**B and E**) or the fetal chamber (**C and F**) the organ-on-a-chip device. Bars indicate mean values ± standard error mean error bars; individual points represent independent biological replicates. *P < 0.05, **P < 0.01, ***P < 0.001, ****P < 0.0001, one-way ANOVA with Tukey’s post hoc multiple corrections test. Fractalkine and sCD40L were significantly induced in gestational membranes and the maternal chamber, but not the fetal chamber of the organ-on-a-chip system in response to GB112 infection.

Infection with GB112 resulted in elevated production of EGF, IL-1α, IL-10, sCD40L, and fractalkine in the maternal and fetal chambers of the organ-on-a-chip models, but not in the primary human gestational membranes (Fig. 6 and 7). Interestingly, GB37 infection did not induce elevated levels of these five cytokines and demonstrated significantly lower cytokine production compared to GB112 in both maternal and fetal chambers of the organ-on-a-chip device as determined by one-way ANOVA with Tukey’s post hoc test (Fig. 6 and 7).

### Gestational membranes challenged with GBS produce IL-15, M-CSF, MCP-3, and MIP-1β, a result not observed in the organ-on-a-chip model

**Fig 8:**
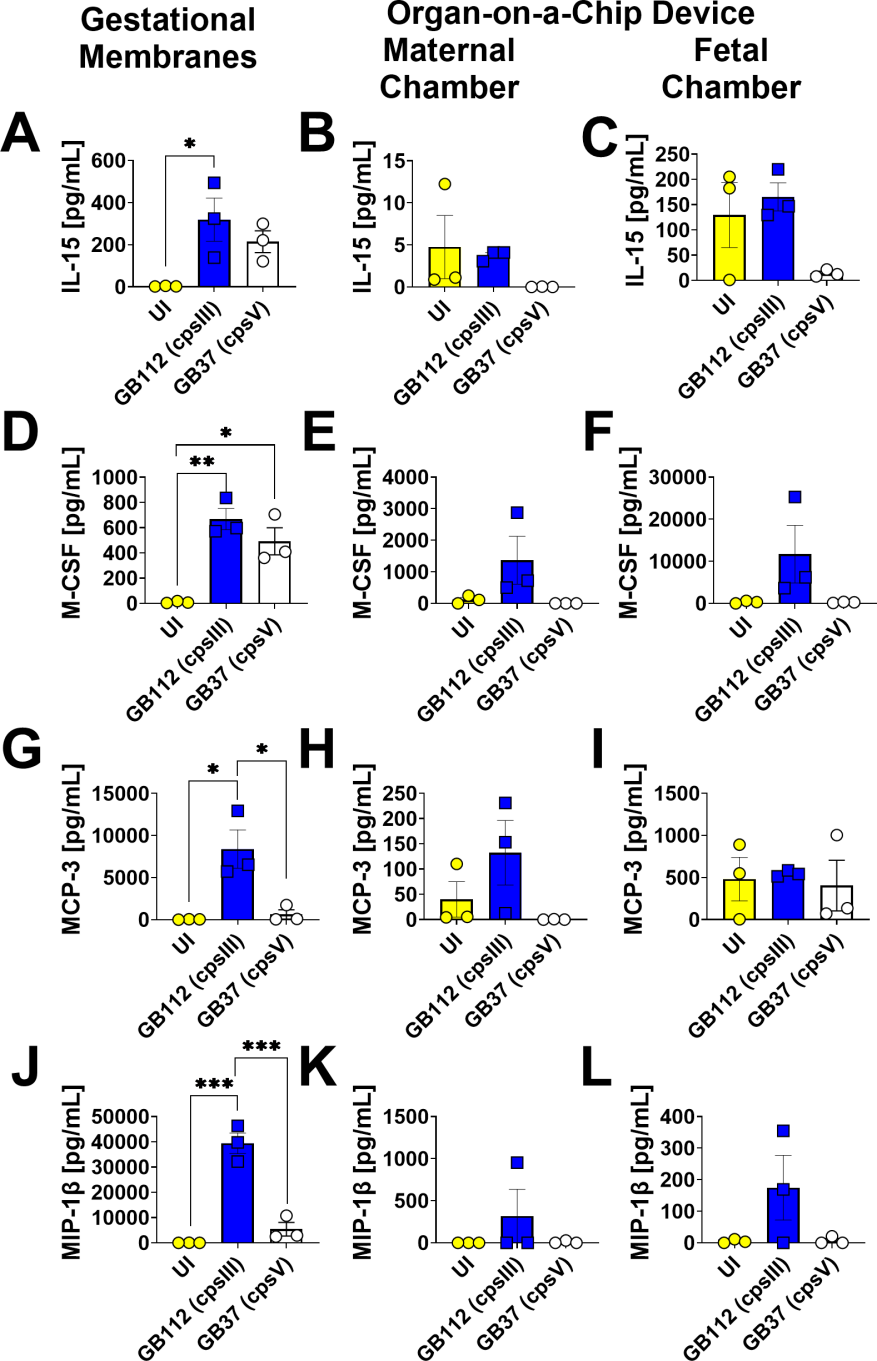
Analysis of IL-15, M-CSF, MCP-3, and MIP-1β production in human gestational membranes or maternal-fetal interface organ-on-a-chip (maternal or fetal chambers). Model systems were either maintained in uninfected conditions (UI, yellow circles and bars) or infected with the perinatal pathogen Group B *Streptococcus* (GB112, blue squares and bars; GB37, white circles and bars). Cytokines including IL-15 (**A–C**), M-CSF (**D–F**), MCP-3 (**G–I**), and MIP-1β (**J–L**) were quantified by multiplex cytokine analyses. Gestational membranes (**A, D, G, J**) are compared to the maternal chamber of (**B, E, H, and K**) or the fetal chamber (**C, F, I, and L**) the organ-on-a-chip device. Bars indicate mean values ± standard error mean error bars; individual points represent independent biological replicates. *P < 0.05, **P < 0.01, ***P < 0.001, ****P < 0.0001, one-way ANOVA with Tukey’s post hoc multiple corrections test. IL-15, M-CSF, MCP-3, and MIP-1β were significantly induced in the human gestational membranes *ex vivo* by GB112 infection but not in the organ-on-a-chip model.

*Ex vivo* infection of human gestational membranes with GB112 resulted in significantly elevated production of IL-15, M-CSF, MCP-3, and MIP-1β—results that were not seen in either the maternal or fetal chamber of the organ-on-a-chip model, as determined by one-way ANOVA with Tukey’s post hoc test (Fig. 8). Conversely, GB37 infection only induced significantly elevated M-CSF compared to uninfected samples in the gestational membranes and did not significantly induce production of IL-15, MCP-3, or MIP-1β in any of the samples tested (Fig. 8).

### GBS infection leads to enhanced production of RANTES, eotaxin, IL-2, IL-5, IL-9, IL-12p40, IL-13, IL-17A, IL-18, IL-22, IFN-α2, and IFN-γ in the maternal chambers of the organ-on-a-chip model, but not the fetal chamber or the gestational membranes *ex vivo*

**Fig 9:**
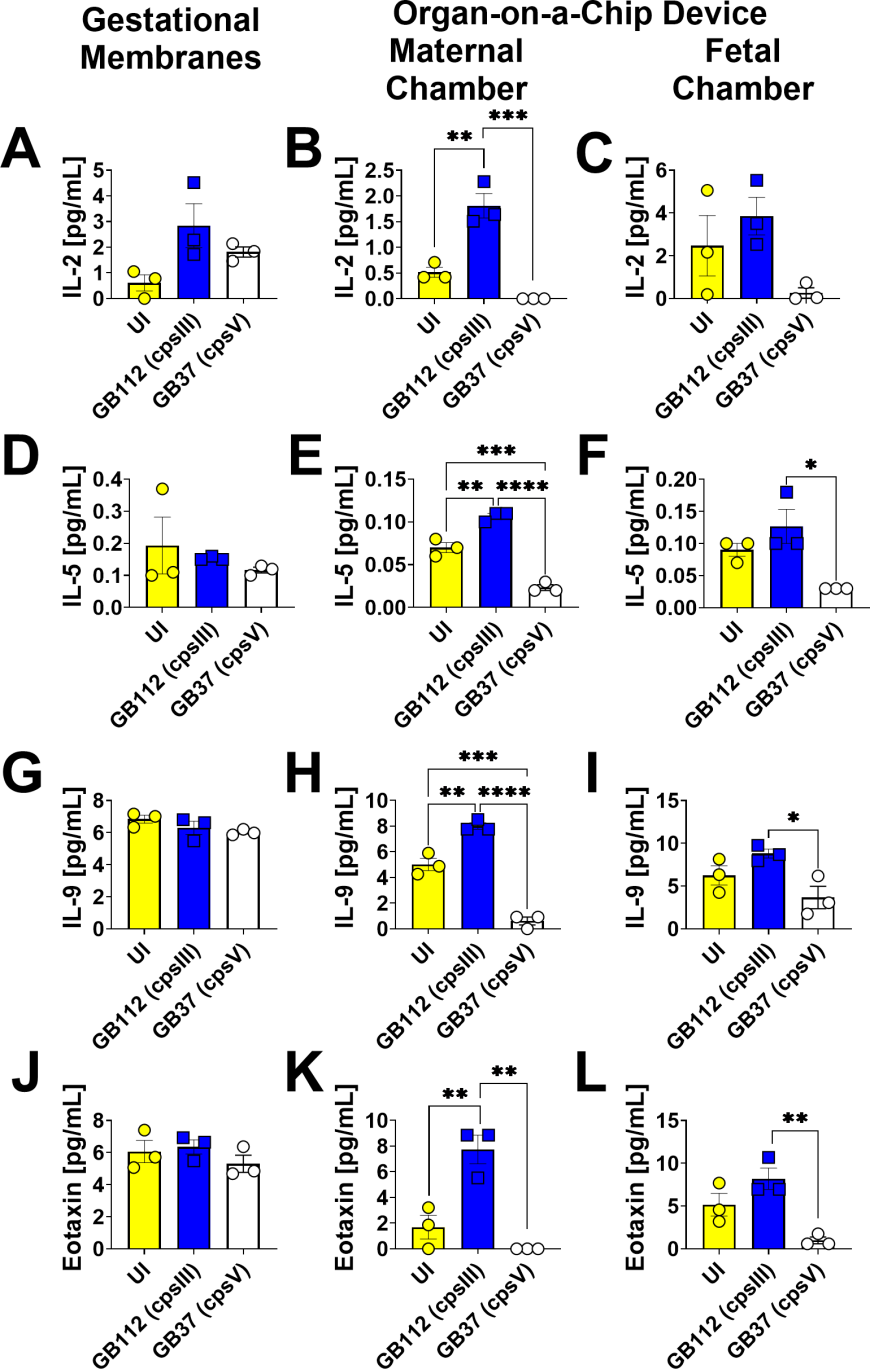
Analysis of IL-2, IL-5, IL-9, and eotaxin production in human gestational membranes or maternal-fetal interface organ-on-a-chip (maternal or fetal chambers). Model systems were either maintained in uninfected conditions (UI, yellow circles and bars) or infected with the perinatal pathogen Group B *Streptococcus* (GB112, blue squares and bars; GB37, white circles and bars). Cytokines including IL-2 (**A–C**), IL-5 (**D–F**), IL-9 (**G–I**), and eotaxin (**J–L**) were quantified by multiplex cytokine analyses. Gestational membranes (**A, D, G, and J**) are compared to the maternal chamber of (**B, E, H, and K**) or the fetal chamber (**C, F, I, and L**) the organ-on-a-chip device. Bars indicate mean values ± standard error mean error bars; individual points represent independent biological replicates. *P < 0.05, **P < 0.01, ***P < 0.001, ****P < 0.0001, one-way ANOVA with Tukey’s post hoc multiple corrections test. MCP-3, MCD, and MIP-1β were significantly induced in the maternal chamber of the organ-on-a-chip device, but not in the fetal chamber or the human gestational membranes *ex vivo* in response to GB112 infection.

**Fig 10:**
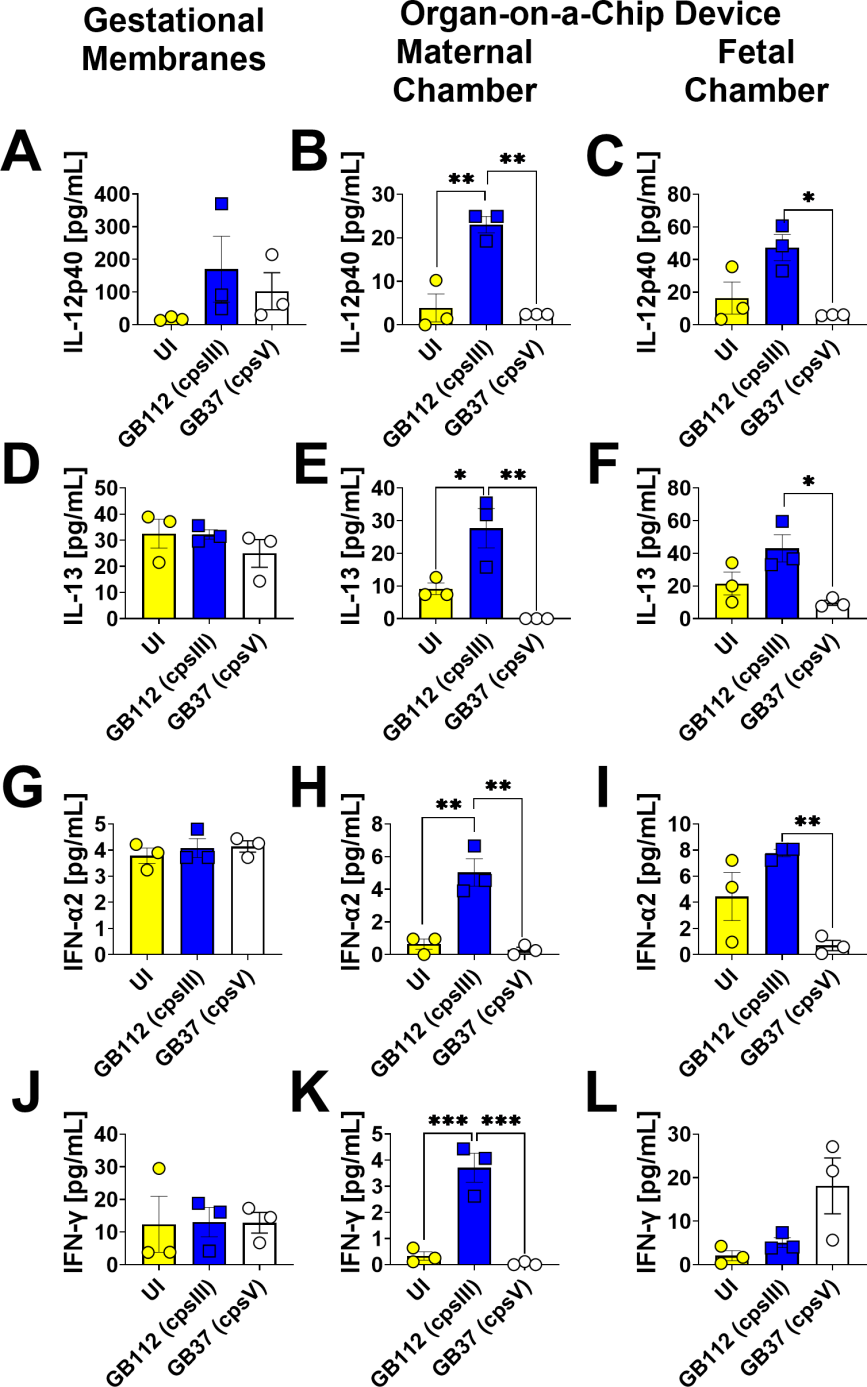
Analysis of IL-12p40, IL-13, IFN-α2, and IFN-γ production in human gestational membranes or maternal-fetal interface organ-on-a-chip (maternal or fetal chambers). Model systems were either maintained in uninfected conditions (UI, yellow circles and bars) or infected with the perinatal pathogen Group B *Streptococcus* (GB112, blue squares and bars; GB37, white circles and bars). Cytokines including IL-12p40 (**A–C**), IL-13 (**D–F**), IFN-α2 (**G–I**), and IFN-γ (**J–L**) were quantified by multiplex cytokine analyses. Gestational membranes (**A, D, G, and J**) are compared to the maternal chamber of (**B, E, H, and K**) or the fetal chamber (**C, F, I, and L**) the organ-on-a-chip device. Bars indicate mean values ± standard error mean error bars; individual points represent independent biological replicates. *P < 0.05, **P < 0.01, ***P < 0.001, ****P < 0.0001, one-way ANOVA with Tukey’s post hoc multiple corrections test. IL-12p40, IL-13, IFN-α2, and IFN-γ were significantly induced in the maternal chamber of the organ-on-a-chip device, but not in the fetal chamber or the human gestational membranes *ex vivo* in response to GB112 infection.

**Fig 11:**
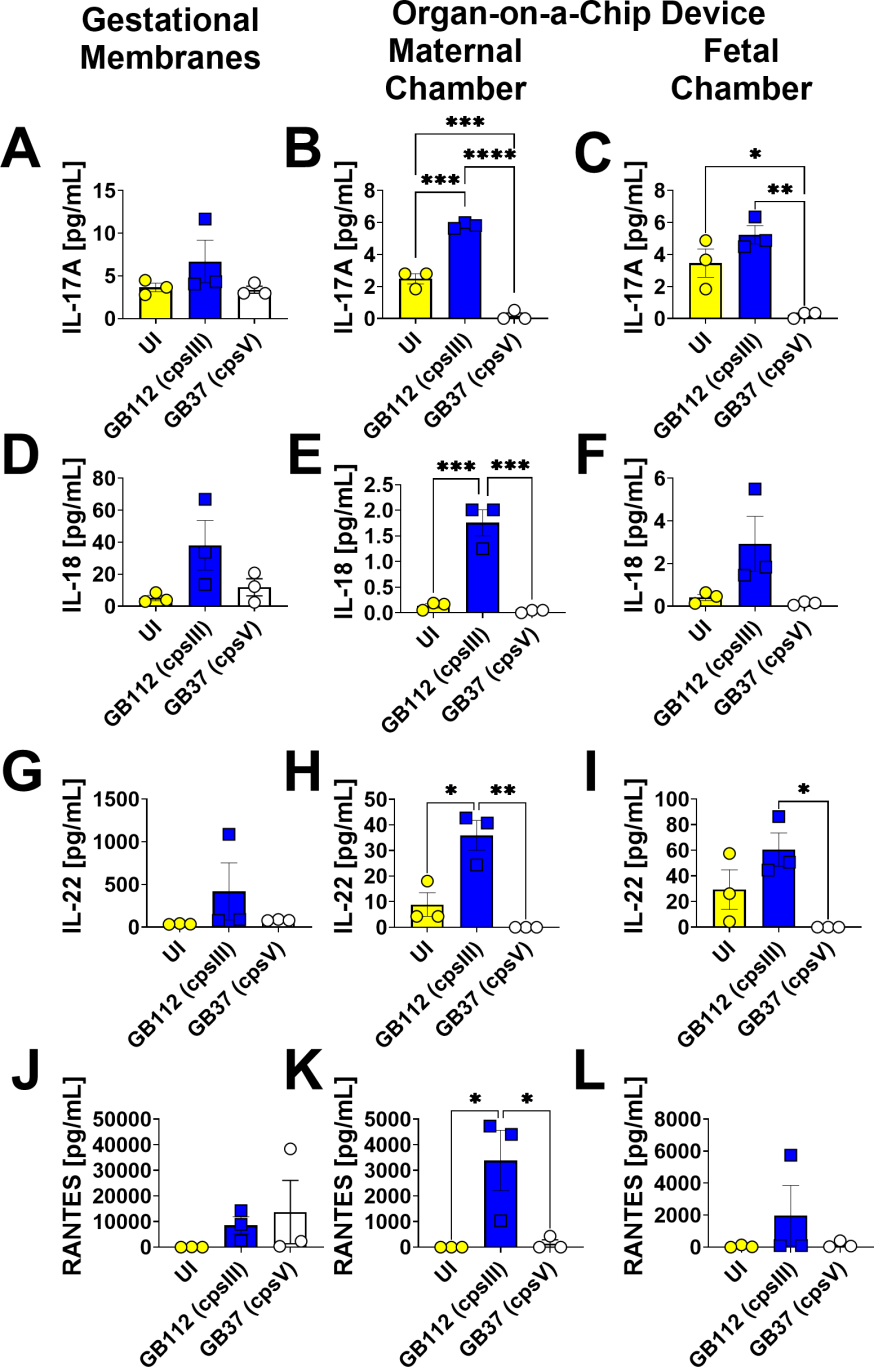
Analysis of IL-17A, IL-18, IL-22, and RANTES production in human gestational membranes or maternal-fetal interface organ-on-a-chip (maternal or fetal chambers). Model systems were either maintained in uninfected conditions (UI, yellow circles and bars) or infected with the perinatal pathogen Group B *Streptococcus* (GB112, blue squares and bars; GB37, white circles and bars). Cytokines including IL-17A (**A–C**), IL-18 (**D–F**), IL-22 (**G–I**), and RANTES (**J–L**) were quantified by multiplex cytokine analyses. Gestational membranes (**A, D, G, and J**) are compared to the maternal chamber of (**B, E, H, and K**) or the fetal chamber (**C, F, I, and L**) the organ-on-a-chip device. Bars indicate mean values ± standard error mean error bars; individual points represent independent biological replicates. *P < 0.05, **P < 0.01, ***P < 0.001, ****P < 0.0001, one-way ANOVA with Tukey’s post hoc multiple corrections test. IL-17A, IL-18, IL-22, and RANTES were significantly induced in the maternal chamber of the organ-on-a-chip device, but not in the fetal chamber or the human gestational membranes *ex vivo* in response to GB112 infection.

GB1112 infection resulted in significantly enhanced production of RANTES, eotaxin, IL-2, IL-5, IL-9, IL-12p40, IL-13, IL-17A, IL-18, IL-22, IFN-α2, and IFN-γ in the maternal chamber. of the organ-on-a-chip, but not the fetal compartment or the primary human gestational membrane model as determined by one-way ANOVA with Tukey’s post hoc test (Fig. 9 to 11). Interestingly, the GB37 strain did not significantly induce RANTES, eotaxin, IL-2, IL-5, IL-9, IL-12p40, IL-13, IL-17A, IL-18, IL-22, IFN-α2, or IFN-γ in the human gestational membranes or the organ-on-a-chip model (Fig. 9 to 11).

### GBS infection leads to enhanced production of FGF in the fetal chamber of the organ-on-a-chip model, but not the maternal chamber or the gestational membranes *ex vivo*

**Fig 12:**
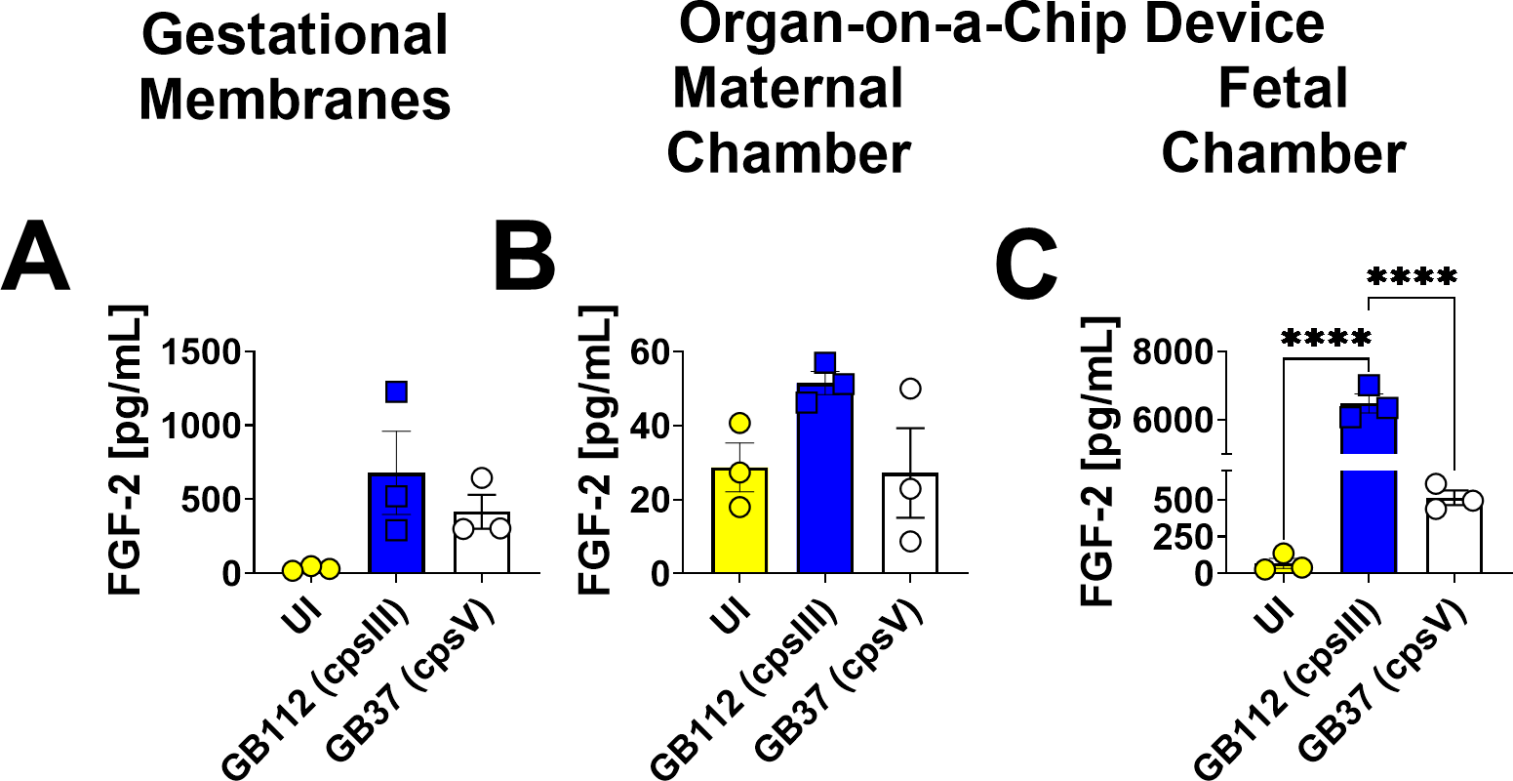
Analysis of FGF-2 production in human gestational membranes or maternal-fetal interface organ-on-a-chip (maternal or fetal chambers). Model systems were either maintained in uninfected conditions (UI, yellow circles and bars) or infected with the perinatal pathogen Group B *Streptococcus* (GB112, blue squares and bars; GB37, white circles and bars). FGF-2 (**A–C**) was quantified by multiplex cytokine analyses. Gestational membranes (**A**) are compared to the maternal chamber of (**B**) or the fetal chamber (**C**) the organ-on-a-chip device. Bars indicate mean values ± standard error mean error bars; individual points represent independent biological replicates. *P < 0.05, **P < 0.01, ***P < 0.001, ****P < 0.0001, one-way ANOVA with Tukey’s post hoc multiple corrections test. FGF-2 was significantly induced in both the fetal and the maternal chamber of the organ-on-a-chip device, but not the human gestational membranes *ex vivo* in response to GB112 infection.

**Fig 13:**
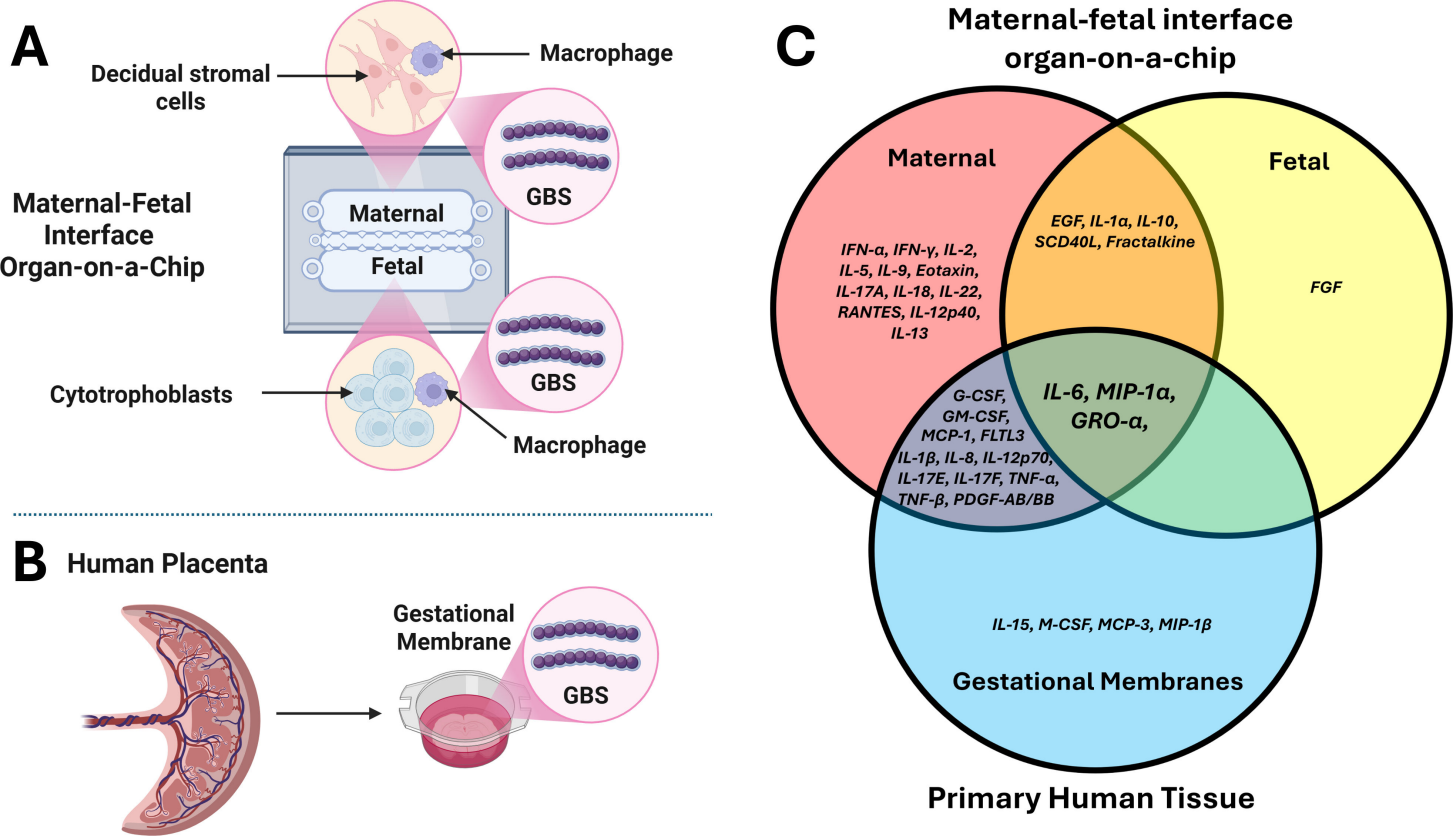
Validation of the maternal-fetal interface organ-on-a-chip with primary human gestational membranes as comparable models to study host immune responses to Group B *Streptococcus* (GBS) infection. Conceptual diagrams of I (**A**) maternal-fetal interface organ-on-a-chip (MFIOC) and I (**B**) human gestational membrane models of GBS infection. (**C**) Multiplex cytokine analyses reveal overlapping cytokine responses between the maternal (pink) and fetal (yellow) compartments of the MFIOC and gestational membranes (light blue) in response to infection with GBS (capsular serotype III strain GB112). Cytokines produced in the maternal compartment and in gestational membranes in response to GBS infection are visualized in the purple region of the Venn diagram. Cytokines produced in the maternal and fetal compartment in response to GBS infection are visualized in the orange region of the Venn diagram. Cytokines produced in maternal and fetal compartments as well as gestational membranes in response to GBS infection are visualized in the center of the Venn diagram.

GBS infection of the maternal-fetal membrane organ-on-a-chip device resulted in an 84% increase in fractalkine (*P* = 0.0123, Student’s *t*-test), an 8-fold increase in IFN-α2 (*P* = 0.0039, Student’s *t*-test), a 50% increase in IL-5 (*P* = 0.0027, Student’s *t*-test), a 60% increase in IL-9 (*P* = 0.0026, Student’s *t*-test), a 2-fold increase in IL-17A (*P* = 0.0003, Student’s *t*-test), a 13-fold increase in IL-18 (*P* = 0.0016, Student’s *t*-test), and a 4-fold increase in IL-22 (*P* = 0.0109, Student’s *t*-test) in the maternal compartment compared to uninfected control samples of the maternal chamber in the organ-on-a-chip (Fig. 12 and 13). Conversely, there were no significant changes in the production of FGF in response to GBS infection in the human gestational membranes *ex vivo* and maternal compartment of the organ-on-a-chip device (Fig. 12).

Interestingly, of the 48 cytokines assessed by multiplex assay, only 11 cytokines (IL-1RA, IL-4, IL-5, IL-7, IL-27, IP-10, MCD, MIG, PDGF-AA, TGF-α, and VEGF) had no significant changes in production in response to GBS infection in the gestational membranes and the organ-on-a-chip models (Fig. S1 to S3).

## DISCUSSION

We have demonstrated here that cytokine responses to a GBS infection in an organ-on-chip model of chorioamnionitis—utilizing human cytotrophoblasts and macrophages to represent the chorion and human decidualized uterine stromal cells and macrophages to represent the decidua—resemble GBS infection of the human choriodecidua *ex vivo* (Fig. 13). This novel dual-chambered organ-on-a-chip system represents the second-generation model system our collaborative team has engineered, building upon the knowledge from our first single chamber organ-on-a-chip system modeling the maternal-fetal interface (31). However, not all results were fully mirrored between the two experimental systems. While the organ-on-chip recapitulated the GRO-α, MIP-1α, and IL-6 responses to infection with the capsular serotype III strain (GB112) in the human choriodecidual punch biopsy samples, other inflammatory mediators had differential expression. Human choriodecidual punch biopsies generated IL-15, IL-27, M-CSF, MCP-3, MDC, and MIP-1β in response to GBS infection, while the organ-on-chip did not. Conversely, the organ-on-chip produced eotaxin, IFN-γ, IL-1α, IL-4, IL-12p40, IL-13, and sCD40L in response to GBS infection, while the human choriodecidual punch biopsies did not.

Additionally, GBS strain-dependent differences were observed across samples infected with the capsular serotype III strain (GB112) and the capsular serotype V strain (GB37). For example, the GB37 strain was attenuated in its ability to induce a variety of cytokines, such as EGF, IL-1α, IL-10, sCD40L, fractalkine, IL-15, MCP-3, MIP-1β, RANTES, eotaxin, IL-2, IL-5, IL-9, IL-12p40, IL-13, IL-17A, IL-18, IL-22, IFN-α2, or IFN-γ, which were significantly induced in a variety of samples by the GB112 strain. Although the underlying mechanism responsible for this difference in cellular responses to GB112 and GB37 is not clear, previous work has shown that immune responses, such as phagocytosis, differ across GBS strain types (32–34). GB37 is phagocytosed to a relatively small degree among the 35 strains tested, while GB112 was phagocytosed to a greater degree (32). The differences in relative inflammation induced by these two strains could possibly be attributed to differences in virulence factors across these strains, including capsule production. GB37 is a capsular serotype V strain, while GB112 is a capsular serotype III strain. Previous work has demonstrated differences in inflammatory pathway activation across capsular serotypes (33). Specifically, sequence type 17 and capsular serotype III strains have been shown to induce enhanced Jun-N-terminal protein kinase (JNK) and nuclear factor κB (NF-κB) pathway activation following GBS infection of macrophages compared with other sequence type or capsular serotype groups (33). These differences in host-pathogen interactions could contribute to alterations in cell signaling pathways that ultimately shape the cytokine and chemokine responses to GBS infection.

Teasing apart the contributions of the maternal and fetal compartments to the immune response against GBS infection, there was enhanced production of many inflammatory mediators (fractalkine, IFN-α2, IL-5, IL-9, IL-17A, IL-18, and IL-22) in the maternal chamber of the organ-on-chip that was not observed from the fetal compartment. These mediators were also not induced from human choriodecidual punch biopsies. The maternal chamber and the gestational membranes additionally responded to GBS infection with enhanced GM-CSF, IL-1β, RANTES, TNF-α, IL-12p70, IL-17E/IL-25, IP-10, MIG, FLT3L, IL-2, and PDGF-AB/BB, while the fetal compartment did not respond. This last instance may be a difference in activation of proinflammatory pathways between maternal and fetal cell types (35).

This work helps elucidate a wide repertoire of cytokines produced at the maternal-fetal interface in response to infection with the perinatal pathogen, GBS. These results are helpful in modeling immune responses to GBS in the gravid reproductive tract that could contribute to adverse perinatal outcomes. Indeed, various studies have shown elevated cytokines which are associated with adverse outcomes. For example, elevated circulating IL-8, IL-12p40, IL-4, IL-13, G-CSF, MIP-1β, and G-MSF levels in pregnant patients were associated with preterm birth (36). In another study, six specific cytokines were significantly high among women with spontaneous preterm birth, including: TNF-α (odds ratio [OR] 3.4, 95% CI [CI] 2.0–6.0, *P* = 0.001), MCP-3 (OR 2.5, 95% CI 1.29–4.67, *P* = 0.007), IL16 (OR 2.4, 95% CI 1.34–4.30, *P* = 0.003), IL-17A (OR 2.3, 95% CI 1.28–4.06, *P* = 0.005), IFN-γ (OR 3.8, 95% CI 2.0–8.0, *P* = 0.001), and fractalkine (OR 2.1, 95% CI 1.17–4.22, *P* = 0.031) (37). IL-15 and MCP-1 have also been shown to be higher in cord blood from preterm births than term births, underscoring a strong association between these cytokines and adverse pregnancy outcomes (38). Another study of cord blood median levels of cytokines associated with significant placental pathology in preterm infants showed elevated eotaxin (*P* = 0.038), G-CSF (*P* = 0.023), IFN-γ (*P* = 0.002), IL-1RA (*P* < 0.001), IL-4 (*P* = 0.005), IL-8 (*P* = 0.010), MCP-1 (*P* = 0.011), and TNF-α (*P* = 0.002) compared to healthy controls. Post hoc analysis revealed sex differences between and within the placental pathology groups (39). Thus, specific types of placental pathology may be associated with differential cytokine induction in sex-dependent fashion that contributes to disease outcomes (39).

Other groups have observed that elevated IL-6, IL-8, M-CSF, and TNF-receptor 2 in the cervicovaginal fluid have been independently associated with acute histological inflammation of the fetal membranes (chorioamnionitis) (40). GM-CSF, IL-15, IL-17, IL-2, IL-2R, VEGF, and MIG concentrations were significantly higher in patients with chorioamnionitis than in those without chorioamnionitis (41). Together, these results demonstrate that a better understanding of the immunological responses to inflammatory insults, such as infection, could lead to better diagnostics and chemotherapeutics that could ameliorate adverse pregnancy outcomes.

It is also important to note that paracrine signaling can occur across the gelatin matrix positioned between our dual chamber system in a diffusion-dependent fashion, including the transfer of small molecules such as cytokines. This undoubtedly affects the responses of neighboring chambers; however, even with this transfer, we were able to detect differential responses in these chambers, underscoring that paracrine signaling plays an important role but is not the only factor contributing to the immunological responses seen in the maternal or fetal compartments.

One limitation of our findings here is that we have only analyzed the cytokine response to GBS infection with two bacterial strains. There are many other ways the choriodecidua can respond to an infection, including the release of lipid mediators of inflammation and parturition, upregulation of membrane-degrading enzymes, stimulation of antimicrobial peptide generation, and recruitment of immune cells, allowing or preventing the formation of biofilms or bacterial invasion of the tissue, which can vary across GBS strains (28, 33, 41–44). Future experiments will test a wider variety of GBS strains across capsular serotypes and isolation sources. Additionally, a future approach will be to use choriodecidual membranes to act as the membrane of a transwell system to assess the contributions of the fetal side of the human membranes *ex vivo* from the maternal side of the membranes, which our organ-on-chip already separates out.

The end goal of building this model is to use each patient’s cells to seed each chamber of the device, including the immune compartment. However, this early work is being done with cell lines as an efficient *in vitro* benchmark to make this technology easier for other laboratories to replicate. By validating and characterizing the model using commercially available cell lines first, we can open the door for this model to groups still navigating administrative hurdles for primary human cell and tissue studies or those lacking consistent access to non-laboring extraplacental tissues. Additionally, our maternal-fetal interface organ-on-a-chip model is accessible to laboratories lacking the resources to simultaneously isolate and culture three primary cell types from human samples, which can be a significant financial, logistic, and technical hurdle.

## SUPPLEMENTAL MATERIAL

Supplemental figures iai.00346-25-s0001.docx Fig. S1 to S3.

ASM does not own the copyrights to Supplemental Material that may be linked to, or accessed through, an article. The authors have granted ASM a non-exclusive, world-wide license to publish the Supplemental Material files. Please contact the corresponding author directly for reuse.
