# Supplementary material for: Comparison of cytokine responses to group B Streptococcus infection in a human maternal-fetal interface organ-on-a-chip system and ex vivo culture model of human gestational membranes
Source: Infect Immun. 2025 Nov 24;93(12):e00346-25. doi: 10.1128/iai.00346-25 (PMC12707140; doi:10.1128/iai.00346-25)
Supplement: Supplemental figures — Fig. S1 to S3. [file iai.00346-25-s0001.docx]

**Supplemental Information**

**Comparison of cytokine responses to Group B *Streptococcus* infection in a human maternal-fetal interface organ-on-a-chip system and *ex vivo* culture model of human gestational membranes**

Leslie A. Kirk^1^, Hannah A. Richards^2^, Danyvid Olivares-Villagómez^1^, Andrea Locke^2,3^, Anthony R. Flores^4^, Shannon D. Manning^5^, David M. Aronoff^6^, Kevin G. Osteen^7,8,9^, David E. Cliffel^2^, Alison J. Eastman^7*^, Jennifer A. Gaddy^1,8,9,10*^

^1^Department of Medicine, Division of Infectious Diseases, Vanderbilt University Medical Center, Nashville, Tennessee, U.S.A.

^2^Department of Chemistry, Vanderbilt University, Nashville, Tennessee, U.S.A.

^3^Department of Biomedical Engineering, Vanderbilt University, Nashville, Tennessee, U.S.A.

^4^Department of Pediatrics, Division of Pediatric Infectious Diseases, Vanderbilt University Medical Center, Nashville, Tennessee, U.S.A.

^5^Department of Microbiology, Genetics, and Immunology, Michigan State University, East Lansing, Michigan, U.S.A.

^6^Department of Medicine, Indiana University School of Medicine, Indianapolis, Indiana, U.S.A.

^7^Department of Obstetrics and Gynecology, Vanderbilt University Medical Center, Nashville, Tennessee, U.S.A.

^8^Department of Pathology, Microbiology, and Immunology, Vanderbilt University Medical Center, Nashville, Tennessee, U.S.A.

^9^Tennessee Valley Health Systems, Department of Veterans Affairs, Nashville, Tennessee, U.S.A.

^10^Medicine Health and Society, Vanderbilt University, Nashville, Tennessee, U.S.A.

*Denotes co-senior authorship

Short Title: Gestational tissue and organ-on-a-chip infection with Group B *Streptococcus*

Address correspondence to:

Jennifer A. Gaddy, Ph.D.

Vanderbilt University Medical Center

Department of Medicine- Division of Infectious Diseases

A2200 Medical Center North

1161 21^st^ Avenue South

Nashville, Tennessee, U.S.A. 37232

Telephone: (615) 873-7884

Fax: (615) 343-6160

[jennifer.a.gaddy@vumc.org](mailto:jennifer.a.gaddy@vumc.org)

-and-

Alison J. Eastman, Ph.D.

Vanderbilt University Medical Center

Department of Obstetrics and Gynecology

S1209 Medical Center North

1161 21^st^ Avenue South

Nashville, Tennessee, U.S.A. 37232

Telephone: (615) 322-4196

[Alison.j.eastman@vumc.org](mailto:Alison.j.eastman@vumc.org)


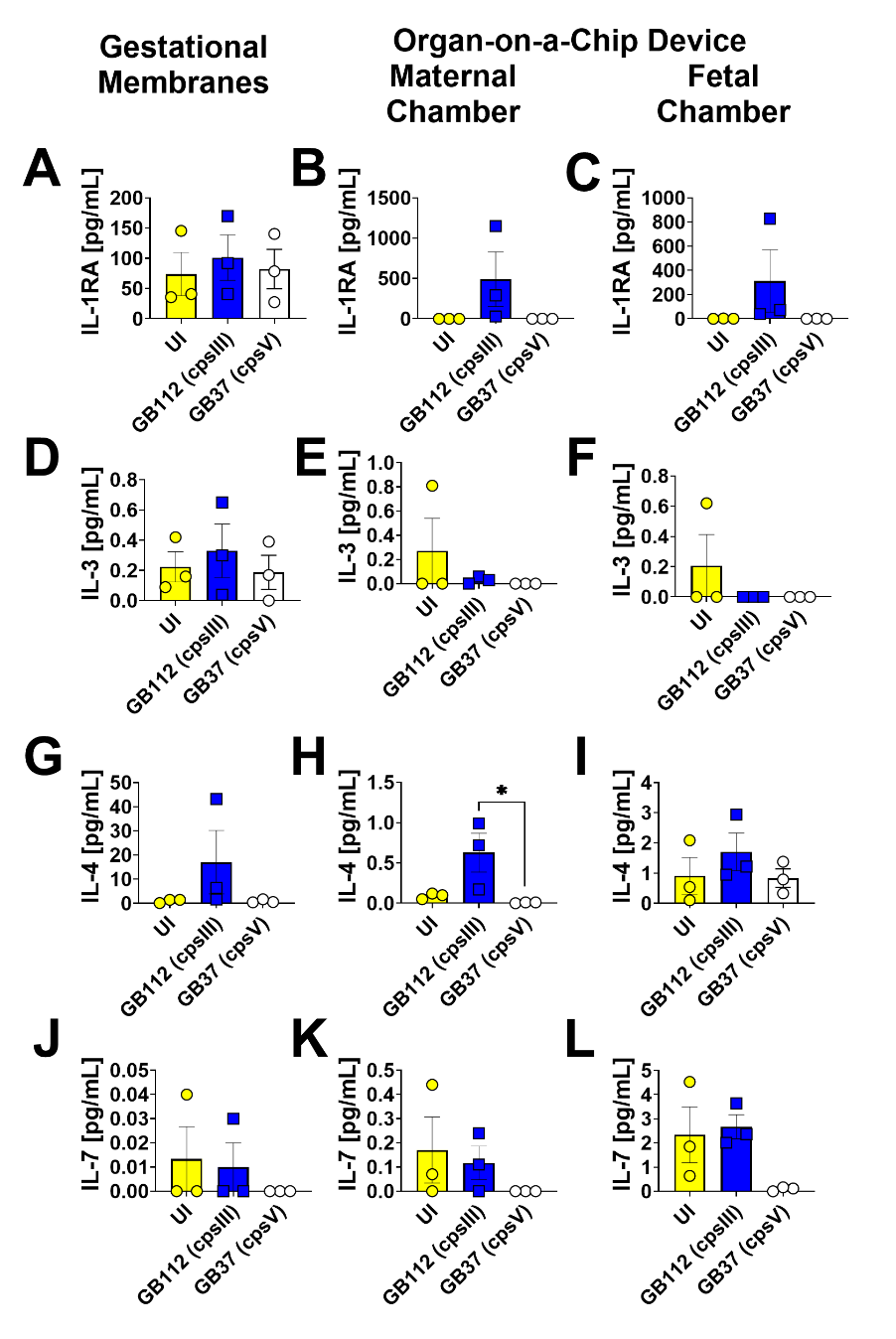


**Supplemental Figure 1**. Analysis of IL-1RA, IL-3, IL-4, and IL-7 production in human gestational membranes (GM) or instrumented maternal-fetal interface organ-on-a-chip (maternal or fetal chambers). Model systems were either maintained in uninfected conditions (UI, yellow circles and bars) or infected with the perinatal pathogen Group B *Streptococcus* (GB112, blue squares and bars, GB37, white circles and bars). Cytokines including IL-1RA (panels A, B, and C), IL-3 (panels D, E, and F), IL-4 (panels G, H, and I), and IL-7 (panels J, K, and L) were quantified by multiplex cytokine analyses. Gestational membranes (panels A, D, G, and J) are compared to the maternal chamber of (panels B, E, H, and K) or the fetal chamber (panels C, F, I, and L) the organ-on-a-chip device. Bars indicate mean values +/- standard error mean error bars, individual points represent independent biological replicates. IL-1RA, IL-3, IL-4, and IL-7 were not significantly induced in response to GBS infection in the gestational membranes or the maternal or fetal chambers of the organ-on-a-chip device in response to GBS infection.


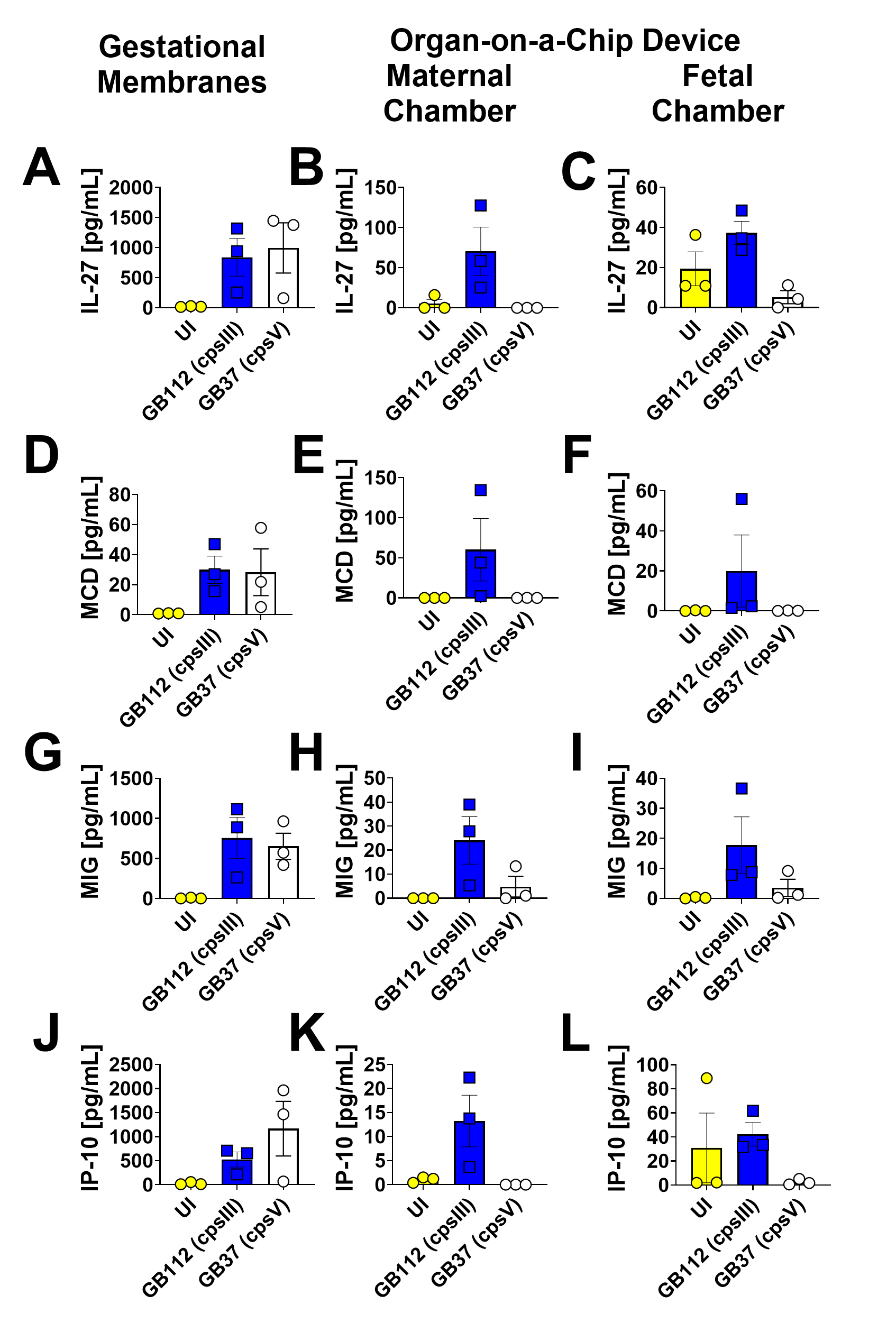


**Supplemental Figure 2**. Analysis of IL-27, MCD, MIG, and IP-10 production in human gestational membranes (GM) or instrumented maternal-fetal interface organ-on-a-chip (maternal or fetal chambers). Model systems were either maintained in uninfected conditions (UI, yellow circles and bars) or infected with perinatal pathogen Group B *Streptococcus* (GB112, blue squares and bars, GB37, white circles and bars). Cytokines including IL-27 (panels A, B, and C), MCD (panels D, E, and F), MIG (panels G, H, and I), and IP-10 (panels J, K, and L) were quantified by multiplex cytokine analyses. Gestational membranes (panels A, D, G and J) are compared to the maternal chamber of (panels B, E, H, and K) or the fetal chamber (panels C, F, I, and L) the organ-on-a-chip device. Bars indicate mean values +/- standard error mean error bars, individual points represent independent biological replicates. IL-27, MCD, MIG, and IP-10 were not significantly induced in response to GBS infection in the gestational membranes or the maternal or fetal chambers of the organ-on-a-chip device in response to GBS infection.


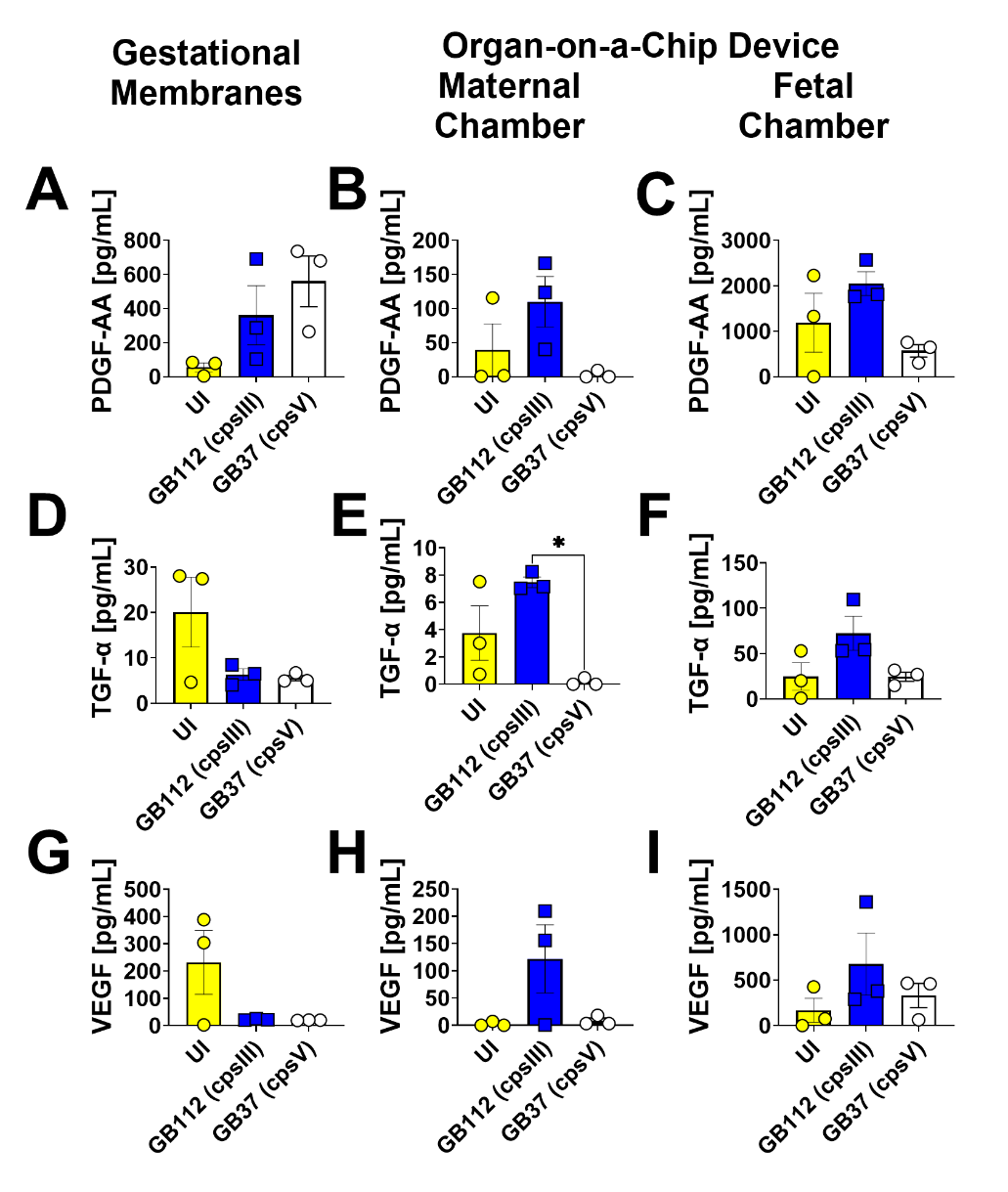


**Supplemental Figure 3**. Analysis of PDGF-AA, TGF-α, and VEGF production in human gestational membranes (GM) or instrumented maternal-fetal interface organ-on-a-chip (maternal or fetal chambers). Model systems were either maintained in uninfected conditions (UI, yellow circles and bars) or infected with the perinatal pathogen Group B *Streptococcus* (GB112, blue squares and bars, GB37, white circles and bars). Cytokines including PDGF-AA (panels A, B, and C), TGF-α (panels D, E, and F), VEGF (panels G, H, and I). were quantified by multiplex cytokine analyses. Gestational membranes (panels A, D, and G) are compared to the maternal chamber of (panels B, E, and H) or the fetal chamber (panels C, F, and I) the organ-on-a-chip device. Bars indicate mean values +/- standard error mean error bars, individual points represent independent biological replicates. PDGF-AA, TGF-α, and VEGF were not significantly induced in response to GBS infection in the gestational membranes or the maternal or fetal chambers of the organ-on-a-chip device in response to GBS infection.
